# Fundamental formulae for wave-energy conversion

**DOI:** 10.1098/rsos.140305

**Published:** 2015-03-18

**Authors:** Johannes Falnes, Adi Kurniawan

**Affiliations:** 1 Department of Physics, Norwegian University of Science and Technology (NTNU), 7491 Trondheim, Norway; 2 School of Marine Science and Engineering, Plymouth University, Plymouth PL4 8AA, UK

**Keywords:** wave-energy conversion, arrays, collective oscillation amplitude, direction-average maximum absorbed power, reactive radiation parameters, kinetic–potential energy difference

## Abstract

The time-average wave power that is absorbed from an incident wave by means of a wave-energy conversion (WEC) unit, or by an array of WEC units—i.e. oscillating immersed bodies and/or oscillating water columns (OWCs)—may be mathematically expressed in terms of the WEC units' complex oscillation amplitudes, or in terms of the generated outgoing (diffracted plus radiated) waves, or alternatively, in terms of the radiated waves alone. Following recent controversy, the corresponding three optional expressions are derived, compared and discussed in this paper. They all provide the correct time-average absorbed power. However, only the first-mentioned expression is applicable to quantify the instantaneous absorbed wave power and the associated reactive power. In this connection, new formulae are derived that relate the ‘added-mass’ matrix, as well as a couple of additional reactive radiation-parameter matrices, to the difference between kinetic energy and potential energy in the water surrounding the immersed oscillating WEC array. Further, a complex collective oscillation amplitude is introduced, which makes it possible to derive, by a very simple algebraic method, various simple expressions for the maximum time-average wave power that may be absorbed by the WEC array. The real-valued time-average absorbed power is illustrated as an axisymmetric paraboloid defined on the complex collective-amplitude plane. This is a simple illustration of the so-called ‘fundamental theorem for wave power’. Finally, the paper also presents a new derivation that extends a recently published result on the direction-average maximum absorbed wave power to cases where the WEC array's radiation damping matrix may be singular and where the WEC array may contain OWCs in addition to oscillating bodies.

## 2. Introduction

For a general three-dimensional case, the basic linearized theory for conversion of ocean-wave energy by means of one oscillating body was developed in the mid-1970s [1–4]. The starting point was to consider power input as the product of the net wave force and the body's oscillation velocity. In addition, Newman [1], based on some reciprocity relations, discussed how the absorbed wave energy is related to wave interference in the far-field region. We may refer to this latter point of view as global, as opposed to local point of view (LPV), which corresponds to the physical process taking place at the immersed oscillating body's wave-interacting surface, i.e. its wetted surface. One purpose of this paper is to compare these two points of view. They are connected through the principle of energy conservation, as well as through a few additional reciprocity relations.

Budal & Falnes [5, p. 478] described, qualitatively, the global point of view (GPV) as follows: ‘a secondary, ring-shaped, outgoing wave is generated, which interferes with the incoming wave in such a way that the resulting transmitted wave carries with it less energy than the incoming wave does’. Subsequently, Budal [6] applied this principle, quantitatively, to discuss wave-energy absorption by an array of oscillating bodies. Some years later, Farley [7] applied a far-field wave-interference analysis to wave-energy conversion by flexible rafts. Contrary to Budal, Farley did not discriminate between two types of outgoing waves, namely diffracted and radiated waves.

After Budal's pioneering work on arrays, Evans [8] and Falnes [9], independently, analysed wave-energy absorption as taking place at the array's wave-interacting surfaces (the bodies' wetted surfaces). Later, this study was extended to include also oscillating water columns (OWCs) in the wave-absorbing array by Falnes & McIver [10] and, independently, by Fernandes [11]. This was also an extension of previous mathematical analyses developed by Falcão & Sarmento [12], Sarmento & Falcão [13] and by Evans [14] for wave-power absorption by OWCs.

Newman [1] presented a review of previously known water-wave reciprocity relations, as well as a few new ones. These reciprocity relations are derived by application of Green's theorem to velocity-potential theory for surface waves on water, which is assumed to be an ideal fluid. Concerning a couple of the presented relations, Newman [1, §7] admitted that they ‘are not physically related to each other in any obvious manner’. Apparently, the application of these relations has caused some controversy recently [15,16] over the question of whether it is the forward (down-wave) or backward (up-wave) radiation that matters. A reason for the controversy may be the existence of at least two versions of what has been called ‘the fundamental theorem for wave power’ [17]. Hopefully, this paper will assist in clarifying the matters discussed.

For any wave-energy converter (WEC) array of oscillating bodies, Wolgamot *et al.* [18] showed that the direction-average maximum absorption width equals *N* times the wavelength divided by 2*π*, on the condition that the array's *N*×*N* radiation-damping matrix is non-singular, where *N* is the array's total number of used oscillating-body modes. In this paper, we generalize this result to cases where the radiation-damping matrix may be singular and where the WEC array may contain OWC units in addition to oscillating bodies. The mathematical details are given in appendix A.

A further subject of this paper is the relationship between the reactive radiation-parameter matrices and the reactive power, which is related to the kinetic–potential energy difference in the water that surrounds the WEC array. It is found that some of the equations which were presented nearly three decades ago by Falnes & McIver [10] need to be corrected.

Throughout this paper, we shall assume that deviation from equilibrium is sufficiently small to make linear theory applicable. We choose a coordinate system with the *z*-axis pointing upwards, where the *z*=0 plane coincides with the mean free surface. We may use Cartesian or polar horizontal coordinates. They are related by (x,y)=(rcos⁡θ,rsin⁡θ). Except for some introductory time-domain consideration, we shall assume an incident, monochromatic, plane wave, for which the wave elevation has a complex amplitude

2.1 $$\eta_{0}=A\;e^{-i(kx\cos\beta +ky\sin\beta )}=A\;e^{-i\{kr\cos(\beta -\theta )\}}$$

(where a time-varying factor e^i*ωt*^ is suppressed). The corresponding incident wave power level (incident wave power transport per unit width of the wave front) is

2.2 $$J_{w}=\frac{\rho gv_{g}|A{|}^{2}}{2},$$

where *A* is the complex wave elevation amplitude of the (undisturbed) incident wave at the origin (*x*,*y*)=(0,0). The incident wave propagates at an angle *β* relative to the *x*-axis. Moreover, *k*=*ω*/*v*_p_ is the angular repetency (wavenumber), *ω* the angular frequency and *v*_p_ the wave's phase velocity. Finally, the wave's group velocity is *v*_g_, the water density is *ρ* and the acceleration of gravity is *g*. Observe that *J*_w_ equals the group velocity multiplied by the propagating incident wave's time-average energy per unit of horizontal sea surface. Half of this energy is potential energy related to water being lifted against gravity from wave troughs to wave crests, while the remaining half is kinetic energy associated with the water's oscillating velocity. However, for a situation where a purely propagating wave, as given by (2.1), interferes with a wave propagating in a different direction, then the surface densities of kinetic energy and potential energy may be different, as discussed in some detail in appendix B.

## 3. Wave-energy absorption at immersed wave-energy converter boundaries

Concerning absorption of wave energy by means of an immersed oscillating body, the instantaneous, as well as the time-average, power absorbed from the wave may be quantified as a product of the net wave force and the velocity of the body. This approach was used, for example, by Budal & Falnes [3] and Evans [2]. There are two contributions to this wave force: firstly, the excitation force, in consequence of the existence of the immersed body, and, secondly, the radiation force, in consequence of the oscillation of the immersed body. The first force contribution is linearly related to the incident wave but independent of the body's motion, while the second force contribution is not explicitly related to the incident wave but linearly related to the body's motion. Assuming, for simplicity, that the immersed WEC body is oscillating in only one mode—mode *i*, say—of its six possible modes (degrees of freedom), then we shall denote the two wave force contributions by *F*_e,*i*,*t*_(*t*) for the excitation force, and by *F*_r,*i*,*t*_(*t*) for the radiation force. In the case of a monochromatic wave and harmonic oscillation with angular frequency *ω*, we denote the complex amplitudes of the two wave-force contributions by

3.1 $$F_{e,i}=f_{e,i}A\;\text{and}\;F_{r,i}=-Z_{ii}u_{i}=-(R_{ii}+i\omega m_{ii})u_{i},$$

respectively, where *u*_*i*_ is the complex velocity amplitude for oscillation mode *i*. The complex proportionality coefficients *f*_e,*i*_=*f*_e,*i*_(*β*,*ω*), i.e. the excitation-force coefficient, and *Z*_*ii*_=*Z*_*ii*_(*ω*), i.e. the radiation impedance, as well as the latter's real and imaginary parts, *R*_*ii*_=*R*_*ii*_(*ω*), i.e. the radiation resistance, and *X*_*ii*_=*ωm*_*ii*_=*X*_*ii*_(*ω*)=*ωm*_*ii*_(*ω*), i.e. the radiation reactance, are functions of *ω*. The coefficient *m*_*ii*_ is called ‘added mass’ although it may be negative in exceptional cases [19]! Moreover, the coefficient *f*_e,*i*_ also depends on *β*, the angle of wave incidence.

Observe that, in terms of complex amplitudes, the radiation-force *F*_r,*i*_ has two components, an active component and a reactive one,

3.2 $$F_{r,i,\text{act}}=-R_{ii}u_{i}\;\text{and}\;F_{r,i,\text{react}}=-iX_{ii}u_{i}=-m_{ii}i\omega u_{i},$$

which are in phase with the velocity *u*_*i*_ and the acceleration i*ωu*_*i*_, respectively. By inverse Fourier transformation, where products in the frequency domain correspond to convolutions in the time domain, we may find a corresponding decomposition of the general, time-domain, radiation force [20]:

3.3 $$F_{r,i,t}(t)=F_{r,i,t,\mathrm{act}}(t)+F_{r,i,t,\mathrm{react}}(t).$$

As shown below, only the active force component contributes to the net time-averaged energy transfer, while the reactive force component serves temporary energy exchange between differently sized stores of kinetic energy and potential energy.

To provide the desired immersed-body motion, the WEC unit needs to be equipped with a machinery for control and power take-off (PTO). This provides an additional force *F*_pto,*i*,*t*_(*t*), with corresponding complex amplitude *F*_pto,*i*_ for the monochromatic-wave case. Then we may write the equation of motion, in complex-amplitude representation, as

3.4 $$\left\{(R_{ii}+r_{\mathrm{loss},i})+i\omega \left(m_{ii}+m_{i}-\frac{c_{i}}{\omega^{2}}\right)\right\}u_{i}=F_{e,i}+F_{\mathrm{pto},i}=f_{e,i}(\beta )A+F_{\mathrm{pto},i},$$

where *m*_*i*_ is the mass of the immersed body and *c*_*i*_ its hydrostatic stiffness coefficient. We have also introduced a coefficient *r*_loss,*i*_ to represent linear power loss. Introducing the body's excursion from equilibrium position *s*_*i*,*t*_(*t*)—thus $u_{i,t}(t)={\dot{s}}_{i,t}(t)$—we may, in time-domain representation, write the equation of motion as

3.5 $$\begin{array}{r} \{-F_{r,i,t,\mathrm{act}}(t)+r_{\mathrm{loss},i}{\dot{s}}_{i,t}(t)\}+\{-F_{r,i,t,\mathrm{react}}(t)+m_{i}{\ddot{s}}_{i,t}(t)+c_{i}s_{i,t}(t)\}=F_{e,i,t}(t)+F_{\mathrm{pto},i,t}(t). \end{array}$$

In contrast to the frequency-domain model, for the time-domain model we may include possible additional nonlinear forces in the *F*_pto,*i*,*t*_(*t*) term of (3.5).

Our next task will be to find an expression for the time-average power *P*_a_ absorbed by the PTO. For this purpose, we multiply through (3.5) by $u_{i,t}(t)={\dot{s}}_{i,t}(t)$, and rearrange terms. We then find

3.6 $$P_{a}\equiv -\bar{F_{\mathrm{pto},i,t}u_{i,t}(t)}=\bar{F_{e,i,t}(t)u_{i,t}(t)}+\bar{F_{r,i,t,\mathrm{act}}(t)u_{i,t}(t)}-r_{\mathrm{loss},i}\bar{{\left\{u_{i,t}(t)\right\}}^{2}},$$

where the overbar denotes averaging over a time interval that is sufficiently long to make the contribution from reactive force components negligible. For periodic waves and oscillations, it is sufficient to average over one period. Note that the reactive-force component—the second one of the two l.h.s. terms of (3.5)—does not contribute to the time-averaged absorbed wave power, as given in (3.6). In relation to *u*_*i*,*t*_(*t*), also *F*_e,*i*,*t*_(*t*) has a reactive part *F*_e,*i*,*t*,react_(*t*), for which $\bar{F_{e,i,t,\mathrm{react}}(t)u_{i,t}(t)}=0$.

The product of the total reactive force and the velocity $u_{i,t}(t)={\dot{s}}_{i,t}(t)$ is the instantaneous reactive power, namely,

3.7 $$\begin{array}{rl} & -F_{e,i,t,\mathrm{react}}(t)u_{i,t}(t)-F_{r,i,t,\mathrm{react}}(t)u_{i,t}(t)+m_{i}{\dot{u}}_{i,t}(t)u_{i,t}(t)+c_{i}s_{i,t}(t){\dot{s}}_{i,t}(t)\\ & \;=\frac{d}{dt}\{W_{i,t,\mathrm{water}}(t)+W_{i,t,\mathrm{body}}(t)\}, \end{array}$$

where $(d/dt)W_{i,t,\mathrm{body}}(t)=m_{i}{\dot{u}}_{i,t}(t)u_{i,t}(t)+c_{i}s_{i,t}(t){\dot{s}}_{i,t}(t)=(d/dt)\{m_{i}u_{i,t}^{2}(t)+c_{i}s_{i,t}^{2}(t)\}/2$ is the time derivative of the sum of the body's kinetic energy and potential energy, and where (d/d*t*)*W*_*i*,*t*,water_(*t*) is the time derivative of the sum of kinetic energy and potential energy of the water surrounding the body. At instants when *u*_*i*,*t*_(*t*)=0, there is no kinetic energy, and at instants when *s*_*i*,*t*_(*t*)=0, there is no potential energy. Except for conditions of resonance, the r.h.s. of (3.7) does not vanish at all instants. In general, the PTO machinery has to cope with reactive forces and reactive power, because of unequal magnitudes of the kinetic and potential energy stores. The r.h.s.—and hence also the l.h.s.—of (3.7) has, however, a vanishing time average.

For a sinusoidal oscillation with complex velocity amplitude $u_{i}=|u_{i}|\exp(i\varphi_{u_{i}})$, we have ${\left\{u_{i,t}(t)\right\}}^{2}=|u_{i}{|}^{2}\cos^{2}(\omega t+\varphi_{u_{i}})=|u_{i}{|}^{2}\{1+\cos(2\omega t+2\varphi_{u_{i}})\}/2$ and ${\left\{s_{i,t}(t)\right\}}^{2}=|u_{i}/\omega {|}^{2}\sin^{2}(\omega t+\varphi_{u_{i}})=|u_{i}/\omega {|}^{2}\{1-\cos(2\omega t+2\varphi_{u_{i}})\}/2$. Using this, we find

3.8 $$\frac{dW_{i,t,\mathrm{body}}(t)}{dt}=\frac{d\{c_{i}s_{i,t}^{2}(t)+m_{i}u_{i,t}^{2}(t)\}/2}{dt}=\left(\frac{c_{i}}{2\omega }-\frac{\omega m_{i}}{2}\right)|u_{i}{|}^{2}\sin(2\omega t+2\varphi_{u_{i}}),$$

which, together with (3.7), explicitly shows how the reactive power is directly related to the difference between the maximum values of kinetic energy and potential energy. In analogy with (3.8), (d/d*t*)*W*_*i*,*t*,water_(*t*) is related to such an energy difference associated with the water surrounding an array of immersed WEC units. This matter is discussed in §6.2,6.3 and more extensively in appendix B.

In the remaining part of this paper, we consider only a monochromatic wave and the corresponding sinusoidal oscillation of immersed WEC units. Without considering the details of the PTO machinery, we shall rather consider the WEC units' complex oscillation amplitudes—for instance *u*_*i*_—to be independent variables, and a goal of our analysis is to find their optimum values corresponding to the incident wave as given by (2.1). Then the complex-amplitude version of (3.6) is

3.9 Pa=Re{−Fpto,iui∗}2=Re{fe,i(β)Aui∗}2−(Rii+rloss,i)uiui∗2,

where the asterisk (*) denotes complex conjugate. Assuming ideal conditions, we set *r*_loss,*i*_=0 in the following.

Moreover, we shall find it convenient to make the following substitutions:

3.10 $$E(\beta )=\frac{f_{e,i}(\beta )u_{i}^{*}}{4}\;\text{and}\;|U{|}^{2}=UU^{*}=\frac{R_{ii}u_{i}u_{i}^{*}}{2}=P_{r},$$

where the non-negative quantity *P*_r_ represents the radiated wave power (caused by any forced oscillation of the immersed body). Although the introduced complex quantity $U=\sqrt{P_{r}}e^{i\delta }$ might, in general, have any arbitrary phase angle *δ* in the interval −*π*<*δ*≤*π*, we shall find it convenient that it is chosen to have the same phase angle as A*E*(*β*). Then A*E*(*β*)/*U* is a real positive quantity, which, notably, is independent of the complex velocity amplitude *u*_*i*_.

We may now simplify (3.9) for the time-averaged absorbed wave power to

3.11 $$P_{a}=P_{e}-P_{r}=AE(\beta )+A^{*}E^{*}(\beta )-|U{|}^{2},$$

where

3.12 $$P_{e}=AE(\beta )+A^{*}E^{*}(\beta )$$

is the ‘excitation power’. An important motivation behind the substitution of $f_{e,i}(\beta )u_{i}^{*}/4$ by *E*(*β*) and $R_{ii}u_{i}u_{i}^{*}/2$ by |*U*|^2^ is that the above (3.11) and (3.12) as well as the following (3.13)–(3.17) are applicable also for a WEC array consisting of several WEC units—oscillating bodies and/or OWCs—provided the parameters *E*(*β*) and |*U*|^2^ are properly redefined, as explained later in this paper (see (6.20) and (7.1) and (7.2)). For this reason, we propose the terms ‘collective excitation-power coefficient’ and ‘collective oscillation amplitude’ for the complex quantities *E*(*β*) and *U*, respectively.

The usefulness of introducing the quantity *U* is that (3.11) may be rewritten as

3.13 $$P_{a}={\left|\frac{AE(\beta )}{U^{*}}\right|}^{2}-{\left|U-\frac{AE(\beta )}{U^{*}}\right|}^{2},$$

from which we, simply by inspection, see that the first term equals the maximum possible absorbed power, provided the last term vanishes, that is, if the quantities *U* and *E*(*β*) have optimum values *U*_0_ and *E*_0_(*β*) that satisfy the optimum condition

3.14 $$U_{0}-\frac{AE_{0}(\beta )}{U_{0}^{*}}=0,\;\text{that is, }\;AE_{0}(\beta )=|U_{0}{|}^{2}=A^{*}E_{0}^{*}(\beta ).$$

Hence, at optimum, the three terms on the r.h.s. of (3.11) have the same, real and non-negative, magnitude. The last one of the three terms is the optimum radiated power. It follows that we have several different alternative expressions for the maximum absorbed power, e.g.

3.15 $$P_{a,\mathrm{MAX}}=P_{r,\mathrm{OPT}}=|U_{0}{|}^{2}=\frac{P_{e,\mathrm{OPT}}}{2}=AE_{0}(\beta )=A^{*}E_{0}^{*}(\beta )=|AE_{0}(\beta )|.$$

We may consider this series of alternative mathematical expressions as reciprocity relations for the maximum absorbed power. For instance, the maximum absorbed power *P*_a,MAX_ equals the optimum radiated power *P*_r,OPT_=|*U*_0_|^2^. In (3.11), the last term, the radiated-power term *P*_r_=|*U*|^2^, appears to be a power-loss term, but it should, rather, be considered as a necessity, because the radiated wave is needed to extract power from—that is, to interfere destructively with—the incident wave.

As we have chosen A*E*(*β*)/*U* to be a real positive quantity, which is independent of *u*_*i*_ and, therefore, also of *U*, we have

3.16 $$\frac{A^{*}E^{*}(\beta )}{U}=\frac{A^{*}E_{0}^{*}(\beta )}{U_{0}}=U_{0}^{*}=U_{0}^{*}(\beta )=|U_{0}(\beta )|=U_{0}(\beta ),$$

where we have made use of the optimum condition (3.14). In general, we shall consider *U* to be an independent complex oscillation-state variable, while the optimum value *U*_0_(*β*) is real and positive, because we have chosen *U* to have the same phase angle as A*E*(*β*) has. According to (3.16), $A^{*}E^{*}(\beta )=U_{0}^{*}U$ and *AE*(*β*)=*U*_0_U*. If we insert this into (3.11) and also use (3.15), we obtain the simple equation

**Figure 1.**
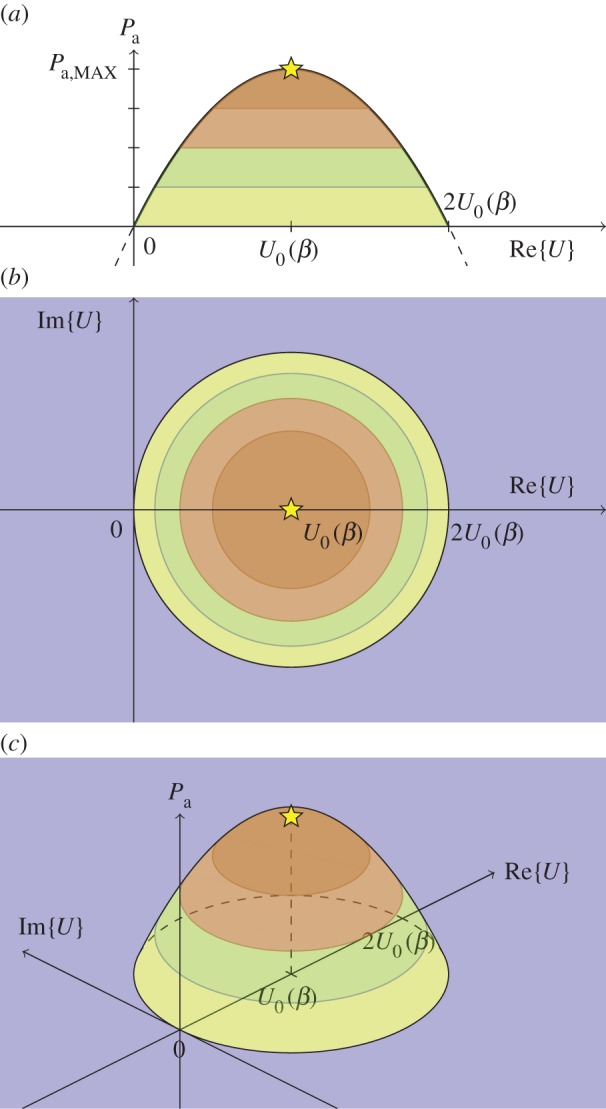
The wave-power ‘island’, illustrating (3.17). Absorbed wave power *P*_a_ as a function of the complex collective oscillation amplitude *U*=Re{*U*}+ i Im{*U*}=|*U*| e^i*δ*^, where the phase δ=arg⁡{U} is chosen such that *AE*/U* is a real positive quantity, and where |*U*| is given by (3.10) for the one-mode oscillating-body case, and by (6.20) for the case of a general WEC array. The largest possible absorbed wave power *P*_a,MAX_ is indicated by a star on the top of the axisymmetric paraboloid, and *U*_0_ is the optimum collective oscillation amplitude. Colour changes indicate levels where *P*_a_/*P*_a,MAX_ equals 0, $\frac{1}{4}$, $\frac{1}{2}$ and $\frac{3}{4}$. (*a*) Side view, (*b*) top view and (*c*) inclined view.

3.17 $$P_{a,\mathrm{MAX}}-P_{a}=U_{0}U_{0}^{*}-U_{0}U^{*}-U_{0}^{*}U+UU^{*}=|U_{0}-U{|}^{2}=|U_{0}(\beta )-U{|}^{2},$$

which, for a fixed value *P*_a_<*P*_a,MAX_, corresponds to the equation of a circle of radius $\sqrt{P_{a,\mathrm{MAX}}-P_{a}}$ centred at *U*_0_(*β*) in the complex *U* plane. Equation (3.17) may be illustrated as an axisymmetric paraboloid in a diagram where a vertical real *P*_a_ axis is erected on a horizontal complex *U* plane, as shown in figure 1.

Assuming that the PTO machinery of the oscillating WEC body contains sufficient control equipment to achieve the desired oscillation, we may consider the complex velocity amplitude *u*_*i*_, as well as *U*, to be an independent variable. However, the optimum value *u*_*i*0_, as well as *U*_0_, depends on the excitation force *f*_e,*i*_(*β*)*A*, and is, consequently, dependent on the incident-wave parameters *A* and *β*. For an optimum oscillation velocity *u*_*i*_=*u*_*i*0_(*β*), say, corresponding to maximum absorbed wave power *P*_a_=*P*_a,MAX_—cf. e.g. (3.14) and (3.15)—we have optimum collective parameters *E*(*β*)=*E*_0_(*β*) and *U*=*U*_0_. Note that |*U*_0_|^2^, as well as all the other alternative expressions given in (3.15) for the maximum absorbed power, depends implicitly on *β*, and, moreover, it is proportional to |*A*|^2^—remembering that *u*_*i*0_(*β*), in contrast to an independent variable *u*_*i*_, is proportional to *A*.

## 4. Destructive far-field wave interference

Excluding waves and oscillations of general time variation, but considering only monochromatic waves and corresponding harmonical oscillations, we may, alternatively, calculate the time-average absorbed power by analysing wave interference in the far-field region, that is, many wavelengths away from the immersed WEC body, or more generally, WEC array. We then have to assume that the wave propagation takes place in water that we may consider to be an ideal loss-free fluid. In the following, we shall apply such a GPV, and then compare the results with the above LPV results.

If we assume that, within a localized area near our chosen origin (*x*,*y*,*z*)=(0,0,0), the body—or, more generally, a WEC array—is installed, but not oscillating, then an incident plane, monochromatic wave, with wave elevation $\eta_{0}=\eta_{0}(r,\theta )=A\;e^{-i\{kr\cos(\beta -\theta )\}}$, as given by (2.1), produces a diffracted wave, for which the wave elevation has a complex amplitude *η*_d_=*η*_d_(*r*,*θ*), say. (This may also include diffraction effects of possible reefs and rocks.) Next, imagine that the immersed body, or the array, is performing forced oscillations with no incident wave, that is, with *A*=0. Then a wave will be radiated from the body, or the array. Let *η*_r_=*η*_r_(*r*,*θ*) denote the complex elevation amplitude of this radiated wave.

When there is an incident wave, and the immersed WEC array is oscillating, then the complex amplitude of the wave elevation is *η*=*η*_0_+*η*_g_=*η*_0_+*η*_d_+*η*_r_. We have here introduced *η*_g_=*η*_d_+*η*_r_ as the complex elevation amplitude of the total ‘outgoing’ wave. Depending on the geometrical details of the array, the outgoing waves may have a complicated mathematical structure near the array, in the so-called near-field region. We shall, however, only need far-field mathematical details, which, in general, are asymptotically valid several wavelengths away from the WEC array. The far-field diffracted/radiated/outgoing wave elevations may thus be expressed in the form

4.1 $$\eta_{d/r/g}=-\frac{i\omega }{g}C_{d/r/g}(\theta ){\left(kr\right)}^{-1/2}\;e^{-ikr}+\cdots ,\;\text{as}\;kr\to \infty ,$$

where the complex functions *C*_d_(*θ*), *C*_r_(*θ*) and *C*_g_(*θ*) are the far-field coefficients for the diffracted wave, the radiated wave and the outgoing wave, respectively. It is convenient to express these coefficients in terms of the so-called Kochin functions

4.2 $$H_{d/r/g}(\theta )=\sqrt{2\pi }C_{d/r/g}(\theta )\;e^{i\pi /4}.$$

Note that the diffracted wave is linearly related to *A*, the complex amplitude of the incident-wave elevation amplitude at the origin, *r*=0, while the radiated wave is linearly related to all WEC units' oscillation amplitudes.

WEC arrays will be discussed in §6. At present, we shall consider the simpler case of only one immersed, single-mode oscillating, body. Introducing complex Kochin function coefficients of proportionality by corresponding lower case symbols, we may write the Kochin functions as

4.3 $$H_{d}(\theta )=h_{d}(\theta )A\;\text{and}\;H_{r}(\theta )=h_{i}(\theta )u_{i}$$

for the diffracted wave and the radiated wave, respectively. The total generated wave's Kochin function is

4.4 $$H_{g}(\theta )=H_{d}(\theta )+H_{r}(\theta ).$$

Note that for an optimum oscillation vector *u*_*i*_=*u*_*i*0_(*β*), there corresponds optimum Kochin functions *H*_r_(*θ*)=*H*_r0_(*θ*) and *H*_g_(*θ*)=*H*_g0_(*θ*), which depend, implicitly, also on *β*. In particular, *u*_*i*0_(*β*) and, thus, *H*_r0_(*θ*) are linearly related to the excitation force *F*_e,*i*_(*β*)=*f*_e,*i*_(*β*)*A*. However, the coefficient *h*_*i*_(*θ*) does not depend on *β*, in contrast to the coefficient *h*_d_(*θ*), which depends implicitly on *β*, since the diffracted wave is a response to the incident wave.

In correspondence with our derivation of (3.9) for the wave power absorbed by an immersed oscillating body as the product of the net wave force and the body's oscillation velocity, Newman [1, §10] expressed the power *P*_a_ absorbed by an oscillating immersed body as an integral over the body's wetted surface, where the integrand is the hydrodynamic pressure multiplied by the normal component of the fluid velocity. Then, applying Green's theorem, he expressed *P*_a_ as an integral over an, envisaged, cylindrical control surface in the far-field region, a surface that encloses the immersed body and all water between the body's wetted surface and the control surface. In this way, Newman [1, eqns 58 and 59] moved from the LPV to the GPV, and expressed the absorbed wave power in terms of Kochin functions.

Accordingly, following Newman, we may write the time-averaged absorbed wave power as

4.5 $$P_{a}=P_{i}-P_{g}=I(\beta )+I^{*}(\beta )-|G{|}^{2}$$

where *P*_i_=*I*(*β*)+I*(*β*) is the ‘input power’ and

4.6 $$P_{g}=|G{|}^{2}=\frac{\omega \rho v_{p}v_{g}}{4\pi g}\int_{0}^{2\pi }|H_{g}(\theta ){|}^{2}\;d\theta =\frac{\omega \rho v_{p}v_{g}}{4\pi g}\int_{0}^{2\pi }|H_{d}(\theta )+H_{r}(\theta ){|}^{2}\;d\theta$$

is the (non-negative) total ‘outgoing power’. Here *v*_p_=*ω*/*k* and *v*_g_=d*ω*/d*k* are the phase velocity and the group velocity, respectively. Moreover,

4.7 $$I(\beta )=\frac{\rho v_{p}v_{g}}{2}H_{g}^{*}(\beta )A=\frac{\rho v_{p}v_{g}}{2}\{H_{d}^{*}(\beta )+H_{r}^{*}(\beta )\}A,$$

and we may write the input power as

4.8 Pi=I(β)+I∗(β)=2Re{I(β)}=ρvpvgRe{Hg∗(β)A}=ρvpvgRe{Hd∗(β)A+Hr∗(β)A}.

An approach corresponding to (4.5)–(4.8) has been applied by Farley [7,15] and Rainey [17]. Their approach shows the physical details of wave-interference energy removal in the far-field region. By wave interference in the far-field region, wave-energy removal takes place where the outgoing wave *η*_g_ travels in the same direction as the incident wave *η*_0_, that is, for direction *θ* coinciding with the incident-wave direction *β*. As there is no energy exchange between two plane waves propagating in different directions, there is no contribution to far-field wave-energy removal by the outgoing wave in directions where *θ*≠*β* (or, more precisely, outside a small *θ* interval around *θ*=*β*, an interval that tends to zero as kr→∞).

Noting that (4.5) has a similar mathematical structure as (3.11), it might appear that (3.14)–(3.17) are valid also if we replace the LPV parameters *P*_e_, *P*_r_, *E*(*β*) and *U* by the GPV parameters *P*_i_, *P*_g_, *I*(*β*)/*A* and *G*, respectively. However, note that the optimum LPV parameters are more directly related to the optimum WEC body oscillations than the GPV ones are. If we compare LPV equations (3.10)–(3.12) with GPV equations (4.5)–(4.8), we may note that *P*_e_ is proportional to *A* and linearly related also to the WEC body's oscillation amplitude, while *P*_r_ is quadratically related to this amplitude, but independent of *A*. By contrast, *P*_i_, as well as *P*_g_, is related in a more complicated way to *A* and to the WEC body's oscillation amplitude. Equations (3.14)–(3.17) therefore do not apply for the GPV parameters.

We may mitigate this drawback by rearranging the GPV equations (4.5)–(4.8) as follows. Firstly, we observe that if the single-mode oscillating WEC body does not oscillate, i.e. *u*_*i*_=0, then no wave energy is being absorbed, i.e. *P*_a_=0. Moreover, the radiated wave's Kochin function vanishes, i.e. *H*_r_(*θ*)=0. Then the GPV equations (4.4)–(4.8) agree with the following reciprocity relation for the diffracted wave's Kochin function [1, eqn 33]:

4.9 $$H_{d}(\beta )A^{*}+H_{d}^{*}(\beta )A=\frac{\omega }{2\pi g}\int_{0}^{2\pi }|H_{d}(\theta ){|}^{2}\;d\theta .$$

Secondly, from the same GPV equations (4.4)–(4.8), we then find, for the oscillating-body case (i.e. *u*_*i*_≠0), that the power *P*_a_, which is removed by the far-field wave interference, is as given by the LPV(!) equations (3.10)–(3.12), but now with collective parameters |*U*|^2^ and *E*(*β*) expressed in terms of far-field quantities, namely,

4.10 $$|U{|}^{2}=\frac{\omega \rho v_{p}v_{g}}{4\pi g}\int_{0}^{2\pi }|H_{r}(\theta ){|}^{2}\;d\theta$$

and

4.11 $$E(\beta )=\frac{\rho v_{p}v_{g}}{2}\left(H_{r}^{*}(\beta )-\frac{\omega }{2\pi g}\int_{0}^{2\pi }\frac{H_{d}(\theta )}{A}H_{r}^{*}(\theta )\;d\theta \right).$$

Note that |*U*|^2^, in contrast to |*G*|^2^, is independent of the wave amplitude *A*, and quadratic in the WEC body's oscillation amplitude. Further, *E*(*β*)*A*, in contrast to *I*(*β*), is linearly related to the oscillation amplitude, and proportional to the incident wave amplitude *A*. Equations (3.10) and (4.10) present two different expressions for the radiated power |*U*|^2^. Physically, this means that the power which is radiated from the WEC body's wave-interacting surface into a lossless fluid equals the power that is associated with the radiated wave in the far-field region of the fluid.

From a physical point of view, what may be observed in the far-field region is a superposition of the plane incident wave and the outgoing wave. As observed in the far-field region, one may not know whether the outgoing wave originates from one single-mode oscillating body or from an array consisting of many WEC units. For this reason, all equations in the present section are valid for this latter WEC system, provided the two-factor product *h*_*i*_*u*_*i*_ that appears in (4.3) is generalized to a sum of such products, one product for each of the WEC array's oscillating modes. Details are given in §6.

## 5. Relationships between radiated and diffracted waves

Among many water-wave reciprocity relations, there are two relations, which relate diffraction and radiation parameters, and about which Newman [1, eqns 45 and 48] remarked that the corresponding two involved physical problems that ‘are not physically related to each other in any obvious manner’. Newman used these two relations to convert the formula for absorbed wave power from the version of (4.5) to the version of (3.11), but with the collective parameters |*U*|^2^ and *E*(*β*) expressed solely in terms of radiation Kochin function coefficients, that is, without the diffraction Kochin function—which we still need to eliminate from (4.11).

The first one of the above-mentioned two reciprocity relations is the Haskind relation [21,22], which relates the excitation force *F*_e,*i*_(*β*)=*f*_e,*i*_(*β*)*A* to the radiated wave's Kochin function *H*_r_(*θ*)|_*θ*=*β*+*π*_=*h*_*i*_(*β* + *π*)*u*_*i*_, namely,

5.1 $$f_{e,i}(\beta )=2\rho v_{p}v_{g}h_{i}(\beta +\pi ).$$

The second one is a relation between *H*_d_(*θ*) and *h*_*i*_(*θ*), a relation which Newman [1, eqn 61] used to simplify (4.11) to

5.2 $$E(\beta )=\frac{\rho v_{p}v_{g}}{2}h_{i}(\beta +\pi )u_{i}^{*}=\frac{\rho v_{p}v_{g}}{2}{\bar{H}}_{r}(\beta +\pi ),$$

where we have introduced the ‘adjoint companion’,

5.3 $${\bar{H}}_{r}(\theta )=h_{i}(\theta )u_{i}^{*},$$

of the radiated wave's Kochin function *H*_r_(*θ*)—cf. (4.3). The complex conjugation star on the complex velocity amplitude *u*_*i*_ in (5.3) corresponds, in time domain, to time-reversed motion.

In this way, Newman succeeded to eliminate, mathematically, the diffracted wave's Kochin function *H*_d_=*h*_d_*A*, that appears for example in (4.5)–(4.8) and in (4.11). Referring to (3.10), we may, however, arrive at the same result (5.2) without referring to the second one of the above-mentioned two, not very obvious, reciprocity relations. The result simply follows by applying the Haskind relation (5.1) to the excitation-force coefficient *f*_e,*i*_(*β*) in (3.10).

Although reciprocity relations between diffraction and radiation parameters connect different physical problems ‘which are not physically related to each other in any obvious manner’, as admitted by Newman [1, §7], the Haskind relation (5.1) may be supported by the following physical argument. Imagine a non-symmetric WEC, e.g. the well-known nodding-duck device [23], which is installed with an optimum orientation to absorb waves arriving from west, thus incident waves propagating eastwards. If, in a case with no incident wave, the device is performing forced oscillations, the device will primarily radiate waves propagating westwards. Thus, the addition of an angle *π* in the argument on the r.h.s. of (5.1) seems reasonable. Moreover, it is reasonable that the excitation-force coefficient *f*_e,*i*_(*β*) of the incident-wave problem (diffraction problem) is proportional to the radiation-ability coefficient *h*_*i*_(*β*+*π*) of the forced-oscillation problem (radiation problem). Admittedly, however, the second one of the two above-mentioned reciprocity relations, which directly connects (4.11) and (5.2), is less obvious from a physical point of view, namely the reciprocity relation presented by Newman [1, eqn 48]:

5.4 $$A{\bar{H}}_{r}(\beta +\pi )=AH_{r}^{*}(\beta )-\frac{\omega }{2\pi g}\int_{0}^{2\pi }H_{d}(\theta )H_{r}^{*}(\theta )\;d\theta .$$

Earlier, the LPV quantities |*U*|^2^ and *E*(*β*) appearing in (3.11) were given by the two equations (3.10). However, when we now have, alternatively, expressed *E*(*β*) by equation (5.2) and |*U*|^2^ by equation (4.10), which are far-field, or global, equations, this corresponds to a mixed, or hybrid, global–local point of view (GLPV), because we have now expressed the LPV parameters |*U*|^2^ and *E*(*β*) in terms of radiation Kochin functions, which are far-field parameters. Although it is not easy to give the GLPV version a direct physical interpretation, it has the advantage that it may be a basis for several reciprocity relations [1] and, moreover, also for certain mathematical derivations below, as exemplified later on in this paper; see §6.1,6.2.

## 6. Generalization to wave-energy converter arrays

**Figure 2.**
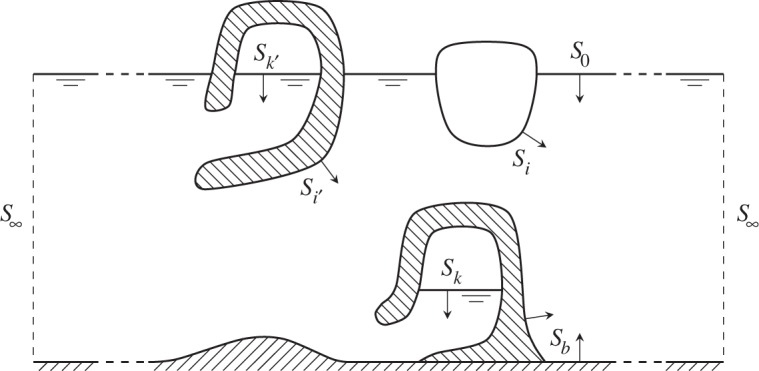
Wave-interacting objects inside an envisaged (control) surface $S_{\infty }$, chosen as a cylindrical surface *r*=const. Two floating bodies are indicated, as well as two OWCs, one in a floating structure, the other in a fixed (bottom-standing) structure. This figure is reproduced from Falnes & Hals [24].

We consider a case of wave-energy absorption by an array of immersed oscillating rigid bodies and of OWCs, as indicated in figure 2. Let us assume that the number of wave-interacting oscillators is *N*=*N*_u_+*N*_p_, where *N*_p_ is the number of OWCs and *N*_u_ is the number of used body modes, whose number may be up to six times the number of bodies. The oscillation state and the excitation due to an incident plane wave may be described by *N*-dimensional column vectors **v** and **x**, respectively, where

6.1 $$\text{v}=\left[\begin{array}{c} \text{u}\\ -\text{p} \end{array}\right]\;\text{and}\;\text{x}=\left[\begin{array}{c} {\text{F}}_{e}\\ -{\text{Q}}_{e} \end{array}\right]=\left[\begin{array}{c} {\text{f}}_{e}\\ -{\text{q}}_{e} \end{array}\right]A.$$

Here we have introduced two *N*_u_-dimensional column vectors **u**=[*u*_1_ *u*_2_ *u*_3_ ⋯ *u*_*N*_u__]^T^ and **F**_e_=[*F*_e,1_ *F*_e,2_ *F*_e,3_ ⋯ *F*_e,*N*_u__]^T^, where *u*_*i*_ and *F*_*i*_ are the complex amplitudes of the oscillation velocity and of the excitation force for rigid-body oscillation mode *i*. Correspondingly, we have introduced two *N*_p_-dimensional column vectors **p**=[*p*_1_ *p*_2_ *p*_3_ ⋯ *p*_*N*_p__]^T^ and **Q**=[*Q*_e,1_ *Q*_e,2_ *Q*_e,3_ ⋯ *Q*_e,*N*_p__]^T^, where *p*_*i*_ and *Q*_*i*_ are the complex amplitudes of the oscillating dynamic air pressure and of the excitation volume flow for OWC *i*. We may think of **v** and **x** as vectors in an *N*-dimensional complex space. The superscript ‘T’ denotes the transpose of a matrix, and the complex conjugate transpose of a matrix is correspondingly denoted by the dagger symbol (†).

Applying linear theory, we have also introduced the following *N*_u_- and *N*_p_-dimensional vectors: the complex vectorial proportionality excitation vector coefficients **f**_e_=**f**_e_(*β*) and **q**_e_=**q**_e_(*β*), respectively. The complex excitation vector **x**=**x**(*β*), acting on the WEC array, depends on the angle *β* of wave incidence, and it is proportional to the complex elevation amplitude *A* of the undisturbed incident wave at the origin (*x*,*y*)=(0,0). We shall, however, here consider the complex oscillation vector **v** to be an independent variable, assuming that we have an ideal machinery for PTO and motion control.

### 6.1 Global point of view

For this, rather general, WEC array, we may extend the second equation of (4.3) to write the radiated wave's Kochin function as

6.2 $$H_{r}(\theta )={\text{v}}^{T}{\text{h}}_{r}(\theta )={\text{h}}_{r}^{T}(\theta )\text{v},\;\text{that is,}\;H_{r}(\theta )=\sum_{i=1}^{N}h_{i}(\theta )v_{i}.$$

We may then generalize *H*_r_ correspondingly in, for example (4.4), (4.6)–(4.8), (4.10) and (4.11). Moreover, the Haskind relation (5.1) is generalized to

6.3 $$\text{x}(\beta )=2\rho v_{p}v_{g}{\text{h}}_{r}(\beta +\pi )A,\;\text{that is},\;x_{i}(\beta )=2\rho v_{p}v_{g}h_{i}(\beta +\pi )A,$$

for *i*=1,2,3,…,*N*. The adjoint radiation Kochin function (5.3) is generalized to [24]

6.4 $${\bar{H}}_{r}(\theta )=\sum_{i=1}^{N}h_{i}(\theta )v_{i}^{*}={\text{h}}_{r}^{T}(\theta ){\text{v}}^{*}={\text{v}}^{†}{\text{h}}_{r}(\theta ),$$

and (5.2) to

6.5 $$E(\beta )=\frac{\rho v_{p}v_{g}}{2}\sum_{i=1}^{N}h_{i}(\beta +\pi )v_{i}^{*}=\frac{\rho v_{p}v_{g}}{2}{\text{v}}^{†}{\text{h}}_{r}(\beta +\pi )=\frac{\rho v_{p}v_{g}}{2}{\bar{H}}_{r}(\beta +\pi ).$$

Note that this latter equation may be considered as a generalized Haskind relation for the collective excitation-power coefficient *E*(*β*). Moreover, on the basis of the general equations (4.11) and (6.5), we easily see that (5.4)—the least obvious one of the reciprocity relations presented by Newman [1, eqn 48]—is still valid with the general radiation Kochin function *H*_r_, as given by (6.2). Equations (4.11) and (6.5) provide two different mathematical relations between the LPV collective excitation-power coefficient *E*(*β*) and the *N* GPV Kochin function coefficients *h*_*i*_ for the radiated wave. Equation (4.10), with (6.2), provides a mathematical relation between these *h*_*i*_ coefficients and the LPV collective amplitude |*U*|, and also the corresponding complex amplitude *U* if we remember that we have chosen the phase of *U* to equal the phase of A*E*(*β*), in accordance with (3.16).

Let us next consider the optimum case for maximum absorbed power. Algebraic procedures for determining the optimum value **v**_0_=[*v*_10_ *v*_20_ *v*_30_ ⋯ *v*_*N*0_]^T^ of the complex oscillation-state vector **v** are treated in more detail in appendix A. Correspondingly, according to (6.2), there exists an optimum Kochin function

6.6 $$H_{r0}(\theta )={{\text{v}}_{0}}^{T}(\beta ){\text{h}}_{r}(\theta )={\text{h}}_{r}^{T}(\theta ){\text{v}}_{0}(\beta ),$$

for the radiated wave. Note that, even if we, in general, consider **v** to be an independent variable, the optimum value **v**_0_=**v**_0_(*β*), as well as the *β*-dependent optimum Kochin function *H*_r0_(*θ*), is linearly related to the incident wave amplitude. From the optimum condition (3.14), we have $AE_{0}(\beta )=|U_{0}{|}^{2}=A^{*}E_{0}^{*}(\beta )$, which, in combination with (4.10) and (4.11), gives the condition

6.7 $$AH_{r0}^{*}(\beta )=\frac{\omega }{2\pi g}\int_{0}^{2\pi }\{H_{d}(\theta )+H_{r0}(\theta )\}H_{r0}^{*}(\theta )\;d\theta ,$$

which the optimum radiated wave's Kochin function *H*_r0_(*θ*) needs to satisfy. Combining this condition with the reciprocity relation (5.4)—see also (6.4)—yields

6.8 $$A{\bar{H}}_{r0}(\beta +\pi )=A{\text{h}}_{r}^{T}(\beta +\pi ){\text{v}}_{0}^{*}(\beta )=\frac{\omega }{2\pi g}\int_{0}^{2\pi }|H_{r0}(\theta ){|}^{2}\;d\theta .$$

That this is real and positive corresponds to the radiated wave having optimum phase. If we choose *A* to be real and positive, then also ${\bar{H}}_{r0}(\beta +\pi )$ has to be real and positive.

In (3.15), we presented several different expressions for the maximum power *P*_a_ that is possible to be absorbed by the WEC array. We shall find it convenient to add also the following expressions:

6.9 $$P_{a,\mathrm{MAX}}=\frac{{\left(P_{a,\mathrm{MAX}}\right)}^{2}}{P_{a,\mathrm{MAX}}}=\frac{{\left(P_{e,\mathrm{OPT}}/2\right)}^{2}}{P_{r,\mathrm{OPT}}}=\frac{{\left\{AE_{0}(\beta )\right\}}^{2}}{|U_{0}{|}^{2}}=\frac{|AE_{0}(\beta ){|}^{2}}{|U_{0}{|}^{2}}.$$

Applying the last one of the fractions shown in the LPV equations (6.9), and then inserting from the GPV equations (4.10) and (6.5), we get

6.10 $$P_{a,\mathrm{MAX}}=\frac{|AE_{0}(\beta ){|}^{2}}{|U_{0}{|}^{2}}=\frac{\rho gv_{g}|A{|}^{2}}{2k}G_{g0}(\beta )=\frac{J_{w}}{k}G_{g0}(\beta )=J_{w}d_{a,\mathrm{MAX}},$$

where *J*_w_ is the wave-power level, as given by (2.2), and *d*_a_≡*P*_a_/*J*_w_ is the ‘absorption width’. Moreover, we have introduced the—at optimum—gain function

6.11 $$G_{g0}(\beta )=\frac{2\pi |{\bar{H}}_{r0}(\beta +\pi ){|}^{2}}{\int_{0}^{2\pi }|H_{r0}(\theta ){|}^{2}\;d\theta }=\frac{2\pi {\text{v}}_{0}^{T}(\beta ){\text{h}}_{r}^{*}(\beta +\pi ){\text{h}}_{r}^{T}(\beta +\pi ){\text{v}}_{0}^{*}(\beta )}{{\text{v}}_{0}^{T}(\beta )\int_{0}^{2\pi }{\text{h}}_{r}(\theta ){\text{h}}_{r}^{†}(\theta )\;d\theta {\text{v}}_{0}^{*}(\beta )},$$

which is an extension of a formula presented, independently, by Newman [25] and by Evans [8] for the single-body, one-mode case. It is remarkable that we here have been able to express the maximum absorbed power in terms of optimum far-field Kochin functions for the radiated wave only. It should be emphasized that this gain function *G*_g0_(*β*) applies only to the optimum case for maximum absorbed wave power.

The Haskind relation (5.2) and the collective Haskind relation (6.5), as well as the optimum gain function as given in (6.11), indicate that it is important for a WEC system to possess the *ability* to radiate a wave propagating in a direction opposite to the direction of the incident wave. This ability is represented, quantitatively, by the *N* coefficients *h*_*i*_(*β*+*π*) in (6.5). If *N*=1, we see from (4.3) and (5.3) that, with *i*=1, we have $|{\bar{H}}_{r}(\beta +\pi ){|}^{2}=|h_{1}(\beta +\pi )v_{1}^{*}{|}^{2}=|h_{1}(\beta +\pi )v_{1}{|}^{2}=|H_{r}(\beta +\pi ){|}^{2}$. However, with (4.3) and (5.3) generalized to (6.2) and (6.4), we should note that, in general, $|{\bar{H}}_{r}(\beta +\pi ){|}^{2}\neq |H_{r}(\beta +\pi ){|}^{2}$ for *N*≥2. For instance, referring to (6.2)–(6.4) for *N*=2, we have, for any *θ*, including *θ*=*β*+*π*, that |H¯r(θ)|2−|Hr(θ)|2=|h1(θ)v1∗+h2(θ)v2∗|2−|h1(θ)v1+h2(θ)v2|2=4 Im{h1(θ)h2∗(θ)} Im{v1v2∗}, which, in general, deviates from zero for arbitrary as well as for optimum values of the complex velocity amplitudes *v*_1_ and *v*_2_. For the circularly oscillating Evans Cylinder [2], we may, as shown below in §7.1—see (7.9)—replace $|{\bar{H}}_{r0}(\beta +\pi ){|}^{2}$ by |*H*_r0_(*β*)|^2^ in the numerator of (6.11). A corresponding replacement may be made if diffraction is negligible or, otherwise, in cases where the integral on the r.h.s. of (5.4) vanishes when *H*_r_(*θ*)=*H*_r0_(*θ*).

Concerning the GPV discussion, presented in §§4 and 5, for the case of one single-mode body WEC unit, we have, so far, here in §6, extended results to the case of an array of WEC units. Our next task will be to generalize some of the LPV matter discussed in §3.

### 6.2 Local point of view

For a single-mode oscillating body, the complex amplitude of two wave-force contributions, the excitation force *F*_e,*i*_ and the radiation force *F*_r,*i*_, are given by (3.1). For our WEC array, the excitation vector **x**, introduced by the second equation of (6.1), is an extension of *F*_e,*i*_, while [10]

6.12 $${\text{x}}_{r}\equiv \left[\begin{array}{c} {\text{F}}_{r}\\ -{\text{Q}}_{r} \end{array}\right]=-\left[\begin{array}{cc} \text{Z} & -\text{H}\\ {\text{H}}^{T} & \text{Y} \end{array}\right]\left[\begin{array}{c} \text{u}\\ -\text{p} \end{array}\right]\equiv -{\text{D}}_{\mathrm{complete}}\text{v}$$

is an extension of *F*_r,*i*_, where **Z** and **Y** are the *N*_u_×*N*_u_ radiation-impedance matrix for the oscillating bodies and the *N*_p_×*N*_p_ radiation-admittance matrix for the OWCs, respectively. These matrices are symmetric, that is, **Z**^T^=**Z** and **Y**^T^=**Y**. The *N*_u_×*N*_p_ matrix **H** represents hydrodynamic coupling between the oscillating bodies and the OWCs, which compose the WEC array. It is convenient to split these complex matrices into real and imaginary parts:

6.13 Z=R+iX,Y=G+iBandH=C+iJ,

where the *radiation resistance matrix* **R**, the radiation reactance matrix **X**, the *radiation conductance matrix* **G**, the radiation susceptance matrix **B**, as well as the matrices **C** and **J**, are real. All these matrices are frequency dependent. Further comments concerning these matrices are given in appendix B; see (B 24)–(B 28) and related text.

Let us now, for the WEC array, extend (3.1) and (3.2), where we defined the radiation force *F*_r,*i*_ and split it into active and reactive components. The extension reads

6.14 $${\text{x}}_{r}=-{\text{D}}_{\mathrm{complete}}\text{v}=-({\text{D}}_{\mathrm{active}}\text{v}+{\text{D}}_{\mathrm{reactive}}\text{v}),$$

where

6.15 $${\text{D}}_{\mathrm{active}}=\left[\begin{array}{cc} \text{R} & -i\text{J}\\ i{\text{J}}^{T} & \text{G} \end{array}\right]\equiv \text{D}\;\text{and}\;{\text{D}}_{\mathrm{reactive}}=i\left[\begin{array}{cc} \text{X} & i\text{C}\\ -i{\text{C}}^{T} & \text{B} \end{array}\right].$$

We may note that

6.16 $$\text{D}={\text{D}}^{†},$$

which means that the *radiation-damping matrix* **D** is hermitian. Also the matrix (**D**_reactive_/i) is hermitian. From this it follows that, for any *N*-dimensional complex column vector **v**, the scalar matrix products **v**^†^**Dv** and **v**^†^**D**_reactive_**v** are real and purely imaginary, respectively. (We may observe that the matrices **D** and (**D**_reactive_/i) are real and symmetric if the WEC array contains no OWCs or no oscillating bodies, that is, in cases where **D**_complete_=**Z**=**R**+i**X** or **D**_complete_=**Y**=**G**+i**B**, respectively.)

If we premultiply (6.14) by −**v**^†^/2, we get the ‘complex radiated power’

6.17 $$P_{r}=-\frac{{\text{v}}^{†}{\text{x}}_{r}}{2}=\frac{{\text{v}}^{†}\text{D}\text{v}}{2}+\frac{{\text{v}}^{†}{\text{D}}_{\mathrm{reactive}}\text{v}}{2},$$

where the last term, the reactive-power term, **v**^†^**D**_reactive_**v**/2 is purely imaginary, while the first term, the radiated-power term, **v**^†^**Dv**/2≡*P*_r_ is real and non-negative—see (6.21). Moreover, if we premultiply by **v**^†^/2 the excitation vector **x**, defined by equation (6.1), we get the ‘complex excitation power’

6.18 $$P_{e}=\frac{{\text{v}}^{†}\text{x}}{2}.$$

We may note that the imaginary part Im{Pe} represents reactive power (see (B 49)).

For any oscillation vector **v**=[**u** −**p**]^T^, the time-average wave power absorbed by the array is *P*_a_=*P*_e_−*P*_r_, where the ‘excitation power’ *P*_e_ and the radiated power *P*_r_ are given by [10]

6.19 Pe=Re{Pe}=v†x+x†v4andPr=Re{Pr}=v†Dv2.

We may express this in the form of (3.11) provided we define the collective excitation-power coefficient *E*(*β*) and the collective oscillation amplitude *U* by

6.20 $$E(\beta )=\frac{{\text{v}}^{†}\text{x}}{4A}\;\text{and}\;|U{|}^{2}=UU^{*}=\frac{{\text{v}}^{†}\text{D}\text{v}}{2},$$

which is an extension of (3.10). We still choose the phase angle of *U* such as to make A*E*(*β*)/*U* a real and positive quantity.

For a case with no incident wave, **x**=**0** (which means that *P*_e_=0), energy conservation requires that the absorbed wave power $P_{a}=P_{e}-P_{r}=-P_{r}=-{\text{v}}^{†}\text{D}\text{v}/2$ cannot be positive. Thus, for all possible finite oscillation-state vectors **v**, we have

6.21 $${\text{v}}^{†}\text{D}\text{v}\geq 0.$$

Thus, in general, the radiation damping matrix **D** is positive semidefinite. It is singular in cases when its determinant vanishes, |**D**|=0. Otherwise, it is positive definite, **v**^†^**Dv**>0.

It is well known [10] that the maximum wave power that can be absorbed by the array is

6.22 $$P_{a,\mathrm{MAX}}=\frac{P_{e,\mathrm{OPT}}}{2}\equiv \frac{{\text{x}}^{†}{\text{v}}_{0}}{4}=\frac{{{\text{v}}_{0}}^{†}\text{x}}{4}=P_{r,\mathrm{OPT}}\equiv \frac{{{\text{v}}_{0}}^{†}\text{D}{\text{v}}_{0}}{2},$$

where **v**_0_=**v**_0_(*β*) is an optimum value of the oscillation-state vector **v** that has to satisfy the optimum condition

6.23 $$\text{D}{\text{v}}_{0}(\beta )=\frac{\text{x}(\beta )}{2}.$$

By manipulating (6.16), (6.19), (6.22) and (6.23), we can show that

6.24 $$P_{a,\mathrm{MAX}}(\beta )-P_{a}=\frac{1}{2}{\left\{\text{v}-{\text{v}}_{0}(\beta )\right\}}^{†}\text{D}\{\text{v}-{\text{v}}_{0}(\beta )\}.$$

For a fixed value of the absorbed wave power *P*_a_, where *P*_a_<*P*_a,MAX_, equation (6.24) represents an ‘ellipsoid’ in the complex *N*-dimensional **v** space, $C^{N}$—but reduced to an *r*_**D**_-dimensional **v** space, $C^{r_{\text{D}}}$, in cases where the radiation damping matrix **D** is singular and of rank *r*_**D**_<*N*—see (A 15). The centre of the ‘ellipsoid’ is at the point **v**=**v**_0_. The elliptical semi-axes are $\sqrt{2(P_{a,\mathrm{MAX}}-P_{a})/\lambda_{i}}$ for *i*=1,2,3,…,*r*_**D**_, where *λ*_*i*_ are the positive definite (non-zero) eigenvalues of the matrix **D**—cf. (A 2). The ‘ellipsoid’ that corresponds to *P*_a_=0 runs through for example points **v**=**0** and **v**=2**v**_0_. The degenerate ‘ellipsoid’ that corresponds to *P*_a_=*P*_a,MAX_ is just one point, which represents the (unconstrained) optimum situation. Choosing smaller *P*_a_ results in increased size of the ‘ellipsoid’. If *N*=1, then the ‘ellipsoid’ simplifies to a circle in the complex *v*_1_ plane. Then, as *v*_10_=*x*_1_/2*D*_11_, we may, from the general equation (6.24), derive |*v*_1_/*v*_10_−1|^2^=(*P*_a,MAX_−*P*_a_)8*D*_11_/|*x*_1_|^2^=1−*P*_a_/*P*_a,MAX_. Note that a similar simple circle equation may be derived for cases where the radiation damping matrix **D** is singular and of rank *r*_**D**_=1, although *N*≥2; as exemplified by (7.25), for an axisymmetric system [26, eqn 37].

Considering how the absorbed power *P*_a_ varies with **v**, the relationship (6.24) may be thought of as a ‘paraboloid’ in the complex *N*-dimensional **v** space, $C^{N}$. The top point of this ‘paraboloid’ corresponds to the optimum, (**v**_0_,*P*_a,MAX_). Here, *N* should be replaced by *r*_**D**_ in cases where the radiation matrix *D* is singular.

The simple equation (3.17), which for a fixed *P*_a_ represents a circle in the complex *U* plane, can be shown to be equivalent to (6.24) above, which represents an ‘ellipsoid’ in the complex **v** space, by making use of (3.16), (6.20) and (6.23). Starting from (3.17), we have

6.25 $$\begin{array}{rl} P_{a,\mathrm{MAX}}-P_{a} & =|U_{0}(\beta )-U{|}^{2}=U_{0}U_{0}^{*}-U_{0}U^{*}-U_{0}^{*}U+UU^{*}\\ & =\frac{1}{2}{\text{v}}_{0}^{†}\text{D}{\text{v}}_{0}-AE-A^{*}E^{*}+\frac{1}{2}{\text{v}}^{†}\text{D}\text{v}\\ & =\frac{1}{2}{\text{v}}_{0}^{†}\text{D}{\text{v}}_{0}-\frac{1}{4}{\text{v}}^{†}\text{x}-\frac{1}{4}{\text{x}}^{†}\text{v}+\frac{1}{2}{\text{v}}^{†}\text{D}\text{v}\\ & =\frac{1}{2}{\text{v}}_{0}^{†}\text{D}{\text{v}}_{0}-\frac{1}{2}{\text{v}}^{†}\text{D}{\text{v}}_{0}-\frac{1}{2}{\text{v}}_{0}^{†}\text{D}\text{v}+\frac{1}{2}{\text{v}}^{†}\text{D}\text{v}\\ & =\frac{1}{2}{\left\{\text{v}-{\text{v}}_{0}(\beta )\right\}}^{†}\text{D}\{\text{v}-{\text{v}}_{0}(\beta )\}, \end{array}$$

noting that the collective excitation-power coefficient *E*(*β*) is a scalar, and thus equals its own transpose, and recalling the hermitian property (6.16) of the radiation-damping matrix **D**.

The proof (6.25) also serves to demonstrate that, with the generalizations (6.20), equations (3.11)–(3.17) are valid not only for a single, one-mode oscillating body, but even for an array consisting of several WEC units—oscillating bodies, as well as OWCs. In particular, equation (3.17), as illustrated in figure 1, is applicable even to the general case of wave energy absorption by an array of oscillating bodies as well as OWCs. The involved physical quantities refer to the array objects' wave-interacting surfaces. Thus, if inverse Fourier transformation is applied to, for example (3.11) and (6.20), they may be applied to analyse the WEC array's wave-power absorption also in the case of non-sinusoidal time variation.

As long as we have not taken any equation of motion into account, we may here consider the components *v*_*i*_ of the vector **v** to be independent variables—assuming that the WEC array contains sufficient control equipment to achieve the desired oscillations. As in the case of a single WEC unit oscillating in one mode (cf. §3), however, all optimum values *v*_*i*0_, and thus the optimum column vector **v**_0_, depend on the excitation vector **x**(*β*), and are, consequently, dependent on the incident-wave parameters *A* and *β*.

### 6.3 Reactive radiation parameters

Let us now return to consider the reactive power corresponding to the radiated wave in a forced oscillation case, that is, without any incident wave. The imaginary part of the complex r.h.s. term in (6.17) is

6.26 $$\frac{1}{2i}{\text{v}}^{†}{\text{D}}_{\mathrm{reactive}}\text{v}=\frac{1}{2}[{\text{u}}^{†}\;-{\text{p}}^{†}]\left[\begin{array}{cc} \text{X} & i\text{C}\\ -i{\text{C}}^{T} & \text{B} \end{array}\right]\left[\begin{array}{c} \text{u}\\ -\text{p} \end{array}\right]=2\omega (T-V),$$

where we, in the last step, made use of (B 25), which applies for a case with no incident wave. Here, *T*−*V* is the time-average difference between kinetic energy and potential energy of the water surrounding the WEC array, or more precisely, the water in the near-field region. (Note that, in the far-field region, such an energy difference averages to zero.) Details are discussed in appendix B.

For the case of a single-mode one-body WEC unit, (3.8) corresponds to a time-domain analogue of the frequency-domain equation (6.26), but with energy difference in the mechanical oscillating system itself, rather than in the surrounding water. For this one-mode case, (6.26) simplifies to

6.27 $$T-V=\frac{X_{ii}|u_{i}{|}^{2}}{4\omega }=\frac{m_{ii}|u_{i}{|}^{2}}{4},$$

where *m*_*ii*_=*m*_*ii*_(*ω*)=*X*_*ii*_(*ω*)/*ω* is the so-called ‘added mass’, a term which may appear confusing in particular cases when it shows up to be negative, that is, in potential-energy-dominating cases where *T*−*V* <0 [19]. From (3.8), we may conclude that the time-average difference between kinetic energy and potential energy of the oscillating body itself is (*m*_*i*_−*c*_*i*_/*ω*^2^)|*u*_*i*_|^2^/4, which is positive only if $\omega >\sqrt{c_{i}/m_{i}}$. Resonance may occur for angular frequencies *ω*=*ω*_0_, which satisfy the equation $\omega_{0}^{2}\{m_{i}+m_{ii}(\omega_{0})\}=c_{i}$. At resonance, the WEC unit's PTO machinery need not exchange reactive power with the oscillating system.

The values of *m*_*ii*_ or, more generally, of **D**_reactive_ at infinite frequency are important for time-domain models. It is well known that the elements of matrix **m**=**X**/*ω*, in general, tend to finite, non-zero, constants at infinite frequency. The infinite-frequency behaviours of the other two matrices which make up the matrix **D**_reactive_ are less well known, although Evans & Porter [27] observed that the radiation susceptance matrix **B** is zero at infinite frequency and Kurniawan *et al.* [28] reported that the real part, **C**, of the radiation coupling matrix has elements which tend to finite, non-zero, constants at infinite frequency. A physical explanation for these behaviours is given in the following.

Consider first an array of oscillating bodies with no OWCs, where one of the bodies is forced to oscillate harmonically with a unit velocity corresponding to mode *i*, in the absence of incident waves, while the other bodies are held fixed. As the oscillation frequency is increased to infinity, the acceleration also increases to infinity. The force required to move the body will necessarily also be infinite. There is therefore sufficient force to accelerate the fluid, which on the wetted body surface needs to move with the same velocity as the body. As the potential energy is zero in this limiting case of infinite frequency (as there are no radiated waves at infinite frequency), while the kinetic energy is positive (as the velocity of the fluid is finite), $m_{ii}(\infty )$ is necessarily positive, according to (6.27). From (B 1), it also follows that m(∞) is positive definite and that the off-diagonal elements of m(∞), i.e. $m_{ij}(\infty )$, are generally non-zero.

Next, consider an array of OWCs with no oscillating bodies, where an oscillating finite pressure is applied on the internal free surface of OWC *i*, in the absence of incident waves, while the other OWCs are open to the atmosphere. As the oscillation frequency is increased to infinity, the force on the free surface remains finite since the pressure is finite. There is therefore insufficient force to accelerate the fluid, and hence the kinetic energy of the fluid is zero. Since the potential energy is also zero at infinite frequency, $B_{ii}(\infty )$ must be zero according to (6.26). It follows that all $B_{ij}(\infty )$ are also zero.

The fact that C(∞) has non-zero elements may be explained by recalling that, in an array of oscillating bodies and OWCs, the radiation coupling coefficient *H*_*ij*_ relates the velocity of rigid-body oscillation mode *j* to the resulting volume flow across the internal free surface of OWC *i*, when it is open to the atmosphere. Since the fluid is assumed to be incompressible, we cannot avoid creating a volume flow by moving the body, even at infinite frequency.

## 7. Two-mode wave-energy converter examples

In agreement with (6.20) we may, for the case of *N*=2 oscillation modes, write the collective excitation-power coefficient as

7.1 $$E(\beta )=\frac{{\text{v}}^{†}\text{x}}{4A}=\frac{v_{1}^{*}x_{1}+v_{2}^{*}x_{2}}{4A},$$

where the complex wave excitation variables *x*_*i*_ and the complex oscillation amplitudes *v*_*i*_ are defined by (6.1). Corresponding to the two r.h.s. terms in (7.1), we may, with reference to (6.4) and (6.5), note that also the adjoint radiation Kochin function ${\bar{H}}_{r}(\theta )$ is, for these examples, composed of two terms. Moreover, the complex collective oscillation amplitude *U* is determined, firstly, by the phase requirement that A*E*(*β*)/*U* is a real and positive quantity, and secondly, by a modulus (magnitude) requirement that

7.2 $$|U{|}^{2}=UU^{*}=\frac{1}{2}{\text{v}}^{†}\text{D}\text{v}=\frac{1}{2}(D_{11}|v_{1}{|}^{2}+D_{12}v_{1}^{*}v_{2}+D_{21}v_{1}v_{2}^{*}+D_{22}|v_{2}{|}^{2}),$$

where the diagonal entries *D*_11_ and *D*_22_ are real, and non-negative. Further, $D_{21}=D_{12}^{*}$ according to the general relation **D**=**D**^†^. Moreover, as is evident from (6.13)–(6.15) and associated text, the off-diagonal entries are either purely imaginary, and thus *D*_21_=−*D*_12_ in the case of one body mode and one OWC mode, or real, and thus *D*_21_=*D*_12_ otherwise.

According to (6.23), the column vector **v**_0_=**v**_0_(*β*), of the optimum complex oscillation amplitudes, has to satisfy the algebraic equation **Dv**_0_=**x**(*β*)/2. This optimum vector **v**_0_ determines the optimum collective parameters *E*_0_(*β*) and *U*_0_. Referring to (3.15), the corresponding maximum absorbed power may be expressed as, for example $P_{a,\mathrm{MAX}}=P_{e,\mathrm{OPT}}/2=AE_{0}(\beta )=P_{r,\mathrm{OPT}}=|U_{0}{|}^{2}={\text{v}}_{0}^{†}\text{D}{\text{v}}_{0}/2$. Considering **v**=[*v*_1_ *v*_2_]^T^ as an independent variable in a two-dimensional complex space $C^{2}$, the relationship

7.3 $$\begin{array}{rl} 2(P_{a,\mathrm{MAX}}-P_{a}) & ={\left({\text{v}}_{0}-\text{v}\right)}^{†}\text{D}({\text{v}}_{0}-\text{v})\\ & =D_{11}|v_{10}-v_{1}{|}^{2}+D_{22}|v_{20}-v_{2}{|}^{2}\\ & \;+D_{12}{\left(v_{10}-v_{1}\right)}^{*}(v_{20}-v_{2})+D_{21}(v_{10}-v_{1}){\left(v_{20}-v_{2}\right)}^{*}, \end{array}$$

see (6.24), represents a ‘paraboloid’ in $C^{2}$, where the top point corresponds to the optimum, **v**=**v**_0_ and *P*_a_=*P*_a,MAX_. See further discussion in §§7.1 and in 8.3.

In the last one of the following three 2-mode examples, §7.1–7.3, which are discussed later, the WEC consists of one OWC and one single-mode oscillating body. Then we set *v*_1_=*u* and *v*_2_=*p*. Moreover, the radiation damping matrix is complex and hermitian, **D**^†^=**D**. In the first two 2-mode examples, only oscillating bodies are involved, and then we set *v*_1_=*u*_1_ and *v*_2_=*u*_2_. Moreover, the radiation damping matrix is real and symmetric, **D**=**R**=**R**^*T*^. In the first example, with one symmetric body in heave and surge, the matrix is diagonal, that is *R*_21_=*R*_12_=0. Then there is no hydrodynamical coupling between the two modes. In the second example, *R*_21_=*R*_12_≠0, and, moreover, *R*_22_=*R*_11_, as we have, for convenience, chosen two equal bodies oscillating in the heave mode, only.

### 7.1 One symmetric body in heave and surge

We shall consider an example with only one immersed body, which has two vertical symmetry planes, one perpendicular to the *x*-axis and one to the *y*-axis. This body is assumed to oscillate in just *N*=2 modes, surge (*i*=1) and heave (*i*=2), with complex velocity amplitudes *v*_1_=*u*_1_ and *v*_2_=*u*_2_, and excitation forces *x*_1_(*β*)=*f*_e,1_(*β*)*A* and *x*_2_(*β*)=*f*_e,2_(*β*)*A*, respectively. The radiated-wave Kochin coefficient is antisymmetric, *h*_1_(*β*+*π*)=−*h*_1_(*β*), for surge, and symmetric, *h*_2_(*β*+*π*)=*h*_2_(*β*), for heave. Because of the body symmetry, the radiation damping matrix is diagonal, i.e. **D**=**R**=diag(*R*_11_,*R*_22_); thus there is no hydrodynamical coupling between the two modes, i.e. *R*_12_=*R*_21_=0. One or both of the diagonal matrix elements *R*_11_ and *R*_22_ for the body may become zero for certain frequencies but are otherwise positive. Let us, however, restrict the following discussion to sufficiently low frequencies to ensure that *R*_11_ and *R*_22_ are never zero, but only positive. Then the radiation damping matrix **D**=**R** is non-singular in the frequency interval of interest.

According to (7.1) and (7.2) we now have, for this symmetric-body example,

7.4 $$E(\beta )=\frac{u_{1}^{*}f_{e,1}(\beta )+u_{2}^{*}f_{e,2}(\beta )}{4}\;\text{and}\;|U{|}^{2}=\frac{R_{11}|u_{1}{|}^{2}+R_{22}|u_{2}{|}^{2}}{2}.$$

It is interesting to note that the last equation here contains two terms, in contrast to the four terms in (7.2). Thus, for this example, (7.4) appears simply as a two-term extension of (3.10). Consequently, because there is no hydrodynamic coupling between the surge and heave modes, the maximum absorbed power may, in agreement with the alternative equations (3.15), be written simply as

7.5 $$|U_{0}{|}^{2}=AE_{0}(\beta )=P_{a,\mathrm{MAX}}=P_{a,\mathrm{MAX}1}+P_{a,\mathrm{MAX}2},$$

where

7.6 $$P_{a,\mathrm{MAX}i}=\frac{R_{ii}|u_{i0}(\beta ){|}^{2}}{2}=\frac{f_{e,i}(\beta )Au_{i0}^{*}(\beta )}{4}=\frac{\rho v_{p}v_{g}h_{i}(\beta +\pi )Au_{i0}^{*}(\beta )}{2},$$

and *u*_*i*0_(*β*) is the optimum value of *u*_*i*_. Here, in the last step, we made use of the Haskind relation (5.1) or (6.3). Using (6.5), we note that *P*_a,MAX_=*AE*_0_(*β*), which is one of the alternative expressions in (3.15).

For this symmetric body, where the radiation-resistance matrix is diagonal, that is **R**=diag(*R*_11_,*R*_22_), the last line in (7.3) vanishes, and thus

7.7 $$2(P_{a,\mathrm{MAX}}-P_{a})={\left({\text{u}}_{0}-\text{u}\right)}^{†}\text{R}({\text{u}}_{0}-\text{u})=R_{11}|u_{10}-u_{1}{|}^{2}+R_{22}|u_{20}-u_{2}{|}^{2}.$$

**Figure 3.**
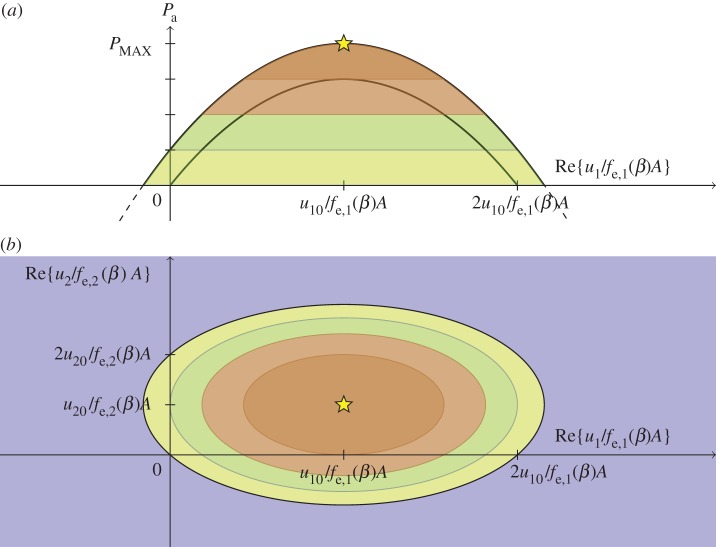
Illustration of equation (7.7) surface cross sections corresponding to Im{*u*_1_/*f*_e,1_(*β*)*A*}=0 and Im{*u*_2_/*f*_e,2_(*β*)*A*}=0. The largest possible absorbed wave power *P*_a,MAX_ is indicated by a star on the top of the paraboloid, and colour changes indicate levels where *P*_a_/*P*_a,MAX_ equals 0, $\frac{1}{4}$, $\frac{1}{2}$ and $\frac{3}{4}$. (*a*) Side view. The upper parabola and the lower parabola are cross sections, of the paraboloid, in the planes Re{*u*_2_/*f*_e,2_(*β*)*A*}=*u*_20_/*f*_e,2_(*β*)*A* and Re{*u*_2_/*f*_e,2_*A*}=0, respectively. (*b*) Top view. The four ellipses indicated by colour changes are, in order of decreasing size, cross sections of the ellipsoids that correspond to *P*_a_/*P*_a,MAX_ equalling 0, $\frac{1}{4}$, $\frac{1}{2}$ and $\frac{3}{4}$, respectively.

For a fixed value of the absorbed wave power *P*_a_, where *P*_a_<*P*_a,MAX_, this equation represents an ‘ellipsoid surface’ in the complex two-dimensional **u** space, $C^{2}$. The centre of the ‘ellipsoid’ is at the point **u**=**u**_0_. The elliptical semi-axes are $\sqrt{2(P_{a,\mathrm{MAX}}-P_{a})/R_{ii}}$ for *i*=1,2. Considering how the absorbed power *P*_a_ varies with **u**, the relationship (7.7) may be thought of as a ‘paraboloid surface’ in the complex two-dimensional **u** space, $C^{2}$. The top point of this ‘paraboloid’ corresponds to the optimum, (**u**_0_,*P*_a,MAX_). If, for a fixed value of *u*_2_, the ‘paraboloid’ is projected onto the complex *u*_1_ plane, this projection corresponds to the axisymmetric surface illustrated in figure 1, but with *P*_a,MAX_−*P*_a_ now replaced by *P*_a,MAX_−*P*_a_−*R*_22_|*u*_20_−*u*_2_|^2^/2, where the last term vanishes if *u*_2_=*u*_20_. Figure 3, on the other hand, indicates what the ‘paraboloid’ looks like, if projected onto a real plane spanned by the Re{*u*_1_/[*f*_e,1_(*β*)*A*]} and Re{*u*_2_/[*f*_e,2_(*β*)*A*]} axes. Graphical illustrations of absorbed wave power, similar to figures 1 and 3, were previously presented by Evans [29].

A particular case of a symmetric body, for which (7.5)–(7.7) apply, is an axisymmetric body, which was analysed by Newman [1, §10], who found

7.8 $$P_{a,\mathrm{MAX}1}=\frac{2J_{w}\cos^{2}\beta }{k}\;\text{and}\;P_{a,\mathrm{MAX}2}=\frac{J_{w}}{k}$$

for this case.

Another particular case of a surging and heaving symmetric body is the famous Evans Cylinder [2]. It is a two-dimensional WEC device, a horizontal circular cylinder, which is submerged below the free water surface. Let the cylinder axis be aligned in the *y*-direction, and let the incident wave propagate in the positive *x*-direction, that is *β*=0. For this submerged cylinder, there is no reflected wave, that is, no wave diffraction in the up-wave direction. This means that the Kochin function for diffraction, as introduced by (4.1)–(4.3), vanishes in the up-wave direction. Hence, it follows from the principle of conservation of energy, that the transmitted wave has the same amplitude |*A*| as the incident wave. Consequently, a non-zero diffracted-wave Kochin function coefficient in the down-wave direction cannot contribute to the amplitude, but only the phase of the transmitted wave.

Another feature of the Evans Cylinder is that the Kochin function coefficients *h*_*i*_, for radiation, as introduced by (6.2), have the property that *h*_1_(*β*)=i*h*_2_(*β*). Thus if we choose *u*_2_=i*u*_1_, which corresponds to a circularly polarized oscillation in the clockwise direction if the *x*-axis is pointing to the right, then *H*_r_(0)=*H*_r_(*β*)=*h*_1_(*β*)*u*_1_+*h*_2_(*β*)*u*_2_=(−ii+1)*h*_2_(*β*)*u*_2_=2*h*_2_(*β*)*u*_2_, while *H*_r_(*π*)=*H*_r_(*β* + *π*)=*h*_1_(*β* + *π*)*u*_1_+*h*_2_(*β*+*π*)*u*_2_= −*h*_1_(*β*)*u*_1_+*h*_2_(*β*)*u*_2_=(ii+1)*h*_2_(*β*)*u*_2_=0. With this circularly polarized oscillation, the radiated waves due to surge and heave are equally large, and they cancel each other in the up-wave direction, but add together constructively in the down-wave direction. Thus, there is neither wave diffraction nor wave radiation in the up-wave direction. All incident wave energy will be absorbed by the Evans Cylinder, provided the circularly polarized oscillation has an optimum amplitude and an optimum phase, in such a way that the down-wave radiated wave exactly cancels the above-mentioned transmitted wave. Half of the incident wave energy is absorbed by each of the two modes, surge and heave.

For the adjoint Kochin function, as defined by (6.4), we now have corresponding expressions, ${\bar{H}}_{r}(0)={\bar{H}}_{r}(\beta )=h_{1}(\beta )u_{1}^{*}+h_{2}(\beta )u_{2}^{*}=(ii+1)h_{2}(\beta )u_{2}^{*}=0=H_{r}(\beta +\pi )=H_{r}(\pi )=H_{r}^{*}(\pi )$, and ${\bar{H}}_{r}(\pi )={\bar{H}}_{r}(\beta +\pi )=h_{1}(\beta +\pi )u_{1}^{*}+h_{2}(\beta +\pi )u_{2}^{*}=-h_{1}(\beta )u_{1}^{*}+h_{2}(\beta )u_{2}^{*}=(-ii+1)h_{2}(\beta )u_{2}^{*}=2h_{2}(\beta )u_{2}^{*}$. We found, above, that *H*_r_(*β*)=*H*_r_(0)=2*h*_2_(*β*)*u*_2_. Thus, we have

7.9 $$|{\bar{H}}_{r}(\beta +\pi )|=|H_{r}(\beta )|=|H_{r}^{*}(\beta )|,$$

if the Evans Cylinder has a circularly polarized oscillation.

### 7.2 Two equal heaving bodies

Let us consider a system of two equal, semisubmerged, axisymmetric bodies with their vertical symmetry axes located at horizontal positions (*x*,*y*)=(∓*d*/2,0). We shall assume that they are oscillating in the heave mode only. With this assumption, the excitation-force vector is of the form **x**(*β*)=**F**_e_(*β*)=[*F*_e,1_(*β*) *F*_e,2_(*β*)]^T^. Further, the radiation damping matrix may be written as

7.10 $$\text{D}=\text{R}=\left[\begin{array}{cc} R_{d} & R_{c}\\ R_{c} & R_{d} \end{array}\right].$$

Note that the diagonal entry *R*_d_ is positive, while the off-diagonal entry *R*_c_, which represents hydrodynamical coupling between the two bodies, may be positive or negative, depending on the distance *d* between the two bodies.

As explained in appendix A.2, we may assume that the matrix **R** is non-singular, and hence $R_{c}^{2}<R_{d}^{2}$. According to (A 10), (A 14), (A 19) and (A 21)–(A 23), the maximum wave power absorbed by the two optimally heaving bodies is

7.11 $$\begin{array}{rl} P_{a,\mathrm{MAX}} & =\frac{|F_{e,1}(\beta )+F_{e,2}(\beta ){|}^{2}}{16(R_{d}+R_{c})}+\frac{|F_{e,1}(\beta )-F_{e,2}(\beta ){|}^{2}}{16(R_{d}-R_{c})}\\ & =\frac{(R_{d}+R_{c})|u_{10}+u_{20}{|}^{2}}{4}+\frac{(R_{d}-R_{c})|u_{10}-u_{20}{|}^{2}}{4} \end{array}$$

and the two bodies' optimum complex velocity amplitudes *u*_10_ and *u*_20_ satisfy

7.12 $$u_{10}+u_{20}=\frac{F_{e,1}(\beta )+F_{e,2}(\beta )}{2(R_{d}+R_{c})}\;\text{and}\;u_{10}-u_{20}=\frac{F_{e,1}(\beta )-F_{e,2}(\beta )}{2(R_{d}-R_{c})}.$$

We observe that (7.11) has a main algebraic structure similar to that of (7.5) and (7.6), which concern example §7.1, where the resistance-damping matrix is diagonal, **R**=diag(*R*_11_,*R*_22_). Before we, in appendix A, derived (7.11) and (7.12) we carried out a similarity transformation in order to diagonalize our given radiation damping matrix (7.10); see the similarity-transforming equations (A 5)–(A 11).

From a wave-body-interaction point of view, it is interesting to note that the first r.h.s. term in (7.11) and the first equation of (7.12) correspond to a sub-optimum situation when the two, equal, heaving bodies cooperate as a source-mode (monopole) radiator, that is, when the constraint *u*_2_=*u*_1_ is applied. Then the two bodies are constrained to heave with equal amplitudes and equal phases. By contrast, the last r.h.s. term in (7.11) and the last equation of (7.12) correspond to a sub-optimum situation when the two bodies are constrained to cooperate as a dipole-mode radiator, that is, when the constraint *u*_2_=−*u*_1_ is applied. In general, (7.11) and (7.12) may be considered to quantify the optimum situation for this combined monopole–dipole wave-absorbing system.

If the maximum radius of each body is sufficiently small, say less than $\frac{1}{30}$ of a wavelength, it may be considered as a point absorber, for which the heave excitation force *F*_e_ is dominated by the Froude–Krylov force, and the diffraction force may be neglected. If, moreover, the centre-to-centre distance *d* between the two bodies is large in comparison with the maximum body radius, then

7.13 $${\text{F}}_{e}=\left[\begin{array}{c} F_{e,1}\\ F_{e,2} \end{array}\right]\approx F_{0}\left[\begin{array}{c} \exp\left\{ik\left(\frac{d}{2}\right)\cos\beta \right\}\\ \exp\left\{-ik\left(\frac{d}{2}\right)\cos\beta \right\} \end{array}\right],$$

where *F*_0_=*σρgπ*[*a*(0)]^2^*A*. Here *a*(0) is each body's water-plane radius, and *A* is the complex amplitude of the incident-wave elevation at the chosen origin (*x*,*y*)=(0,0). Further, *σ*≤1 is a factor that corrects for the diminishing of hydrodynamic pressure with distance below the water surface. (In many cases of practical interest, this correction factor may be approximated to *σ*≈1.) For this point-absorber case, the entries in the radiation-resistance matrix **R** in (7.10) are approximately given by [9, eqns 43–44] (see also (A 8))

7.14 $$R_{d}\approx R_{0}=\frac{k|F_{0}{|}^{2}}{8J_{w}}=\frac{k|F_{0}/A{|}^{2}}{4\rho gv_{g}}\;\text{and}\;R_{c}\approx R_{0}J_{0}(kd),$$

where *J*_0_ denotes the Bessel function of the first kind and zero order. We observe that the matrix **R** is non-singular, and moreover,

7.15 $$R_{d}+R_{c}\approx R_{0}(1+J_{0}(kd))\;\text{and}\;R_{d}-R_{c}\approx R_{0}(1-J_{0}(kd))$$

are positive, since −1<*J*_0_(*kd*)<1 for *kd*>0.

Using formulae (7.11)–(7.15), we find

7.16 $$\left[\begin{array}{c} F_{e,1}+F_{e,2}\\ F_{e,1}-F_{e,2} \end{array}\right]\approx 2F_{0}\left[\begin{array}{c} \cos\left\{\frac{kd}{2}\cos\beta \right\}\\ i\sin\left\{\frac{kd}{2}\cos\beta \right\} \end{array}\right]$$

and

7.17 $$\begin{array}{rl} P_{a,\mathrm{MAX}}(\beta ) & \approx \frac{|F_{0}{|}^{2}}{4R_{0}}\left(\frac{\cos^{2}\{k(d/2)\cos\beta \}}{1+J_{0}(kd)}+\frac{\sin^{2}\{k(d/2)\cos\beta \}}{1-J_{0}(kd)}\right)\\ & =\frac{|F_{0}{|}^{2}}{4R_{0}}\frac{1-J_{0}(kd)\cos\{kd\cos\beta \}}{1-J_{0}^{2}(kd)}. \end{array}$$

Note that, in general, this maximum absorbed power is not equally divided between these two bodies [9, eqn 51].

We may note from the point-absorber approximation (7.13) that, since *F*_0_/*A* is real, $f_{e,i}(\beta )=F_{e,i}(\beta )/A=F_{e,i}^{*}(\beta +\pi )/A^{*}=f_{e,i}^{*}(\beta +\pi )$ for *i*=1,2. Correspondingly, we then find from (6.2)–(6.4) that ${\bar{H}}_{r}(\beta )=h_{1}(\beta )u_{1}^{*}+h_{2}(\beta )u_{2}^{*}=h_{1}^{*}(\beta +\pi )u_{1}^{*}+h_{2}^{*}(\beta +\pi )u_{2}^{*}=H_{r}^{*}(\beta +\pi )$ and, similarly, ${\bar{H}}_{r}(\beta +\pi )=H_{r}^{*}(\beta )$. Thus, for the two considered heaving bodies, we have here explicitly demonstrated that the term containing the integral on the r.h.s. of (5.4) is, as expected, negligible in the point-absorber limit, because diffraction effects are then negligible.

### 7.3 One single-mode body and one oscillating water column

We consider one single floating body that contains one OWC, and we make the simplifying assumption that only one rigid-body oscillating mode is involved. It could be, for instance, a BBDB device structure [30], in a case where the OWC-containing body is restricted to oscillate in the pitch mode only. Otherwise, we shall also discuss an axisymmetric system where the rigid-body structure is restricted to oscillate in the heave mode only.

With this example, the two *N*-dimensional column vectors **v** and **x**, as well as the *N*×*N* radiation damping matrix **D**, introduced by (6.1), as well as (6.15), reduce to the following two two-dimensional vectors:

7.18 $$\text{v}=\left[\begin{array}{c} u\\ -p \end{array}\right]\;\text{and}\;\text{x}=\left[\begin{array}{c} F_{e}\\ -Q_{e} \end{array}\right],$$

as well as the 2×2 matrix

7.19 $$\text{D}=\left[\begin{array}{cc} R & -iJ\\ iJ & G \end{array}\right],$$

respectively.

In order to determine the maximum absorbed power and the corresponding optimum oscillation, it is convenient to apply similarity transformation as shown in some detail in appendix A. For the present example, the eigenvalues *λ*_1_ and *λ*_2_ of the radiation damping matrix (7.19) are solutions of the second-degree algebraic equation |**D**−*λ***I**|=*λ*^2^−(*R*+*G*)*λ*+*RG*−*J*^2^=0. Thus, *λ*_1_ and *λ*_2_ are given by

7.20 $$\lambda_{i}=\frac{R+G-{\left(-1\right)}^{i}\sqrt{{\left(R+G\right)}^{2}-4(RG-J^{2})}}{2}\;\text{for }i=1,2.$$

The corresponding two eigenvectors, which satisfy (A 2) and (A 4), are

7.21 $${\text{e}}_{i}=C_{i}\left[\begin{array}{c} iJ\\ R-\lambda_{i} \end{array}\right],\;\text{where}\;C_{i}=\frac{1}{\sqrt{{\left(R-\lambda_{i}\right)}^{2}+J^{2}}}.$$

In terms of similarity transformed excitation amplitudes *x*′_*i*_(*β*) and corresponding optimum oscillation amplitudes *v*′_*i*0_(*β*), both of which are given below, the maximum absorbed power may, according to (A 10) and (A 11), be written as

7.22 $$P_{a,\mathrm{MAX}}=\sum_{i=1}^{2}\frac{|x_{i}^{\prime }(\beta ){|}^{2}}{8\lambda_{i}}=\frac{1}{2}\sum_{i=1}^{2}\lambda_{i}|v_{i0}^{\prime }(\beta ){|}^{2}=\frac{1}{2}\lambda_{1}|v_{10}^{\prime }(\beta ){|}^{2}+\frac{1}{2}\lambda_{2}|v_{20}^{\prime }(\beta ){|}^{2},$$

corresponding to the optimum condition

7.23 $$\lambda_{i}v_{i0}^{\prime }(\beta )=\frac{1}{2}x_{i}^{\prime }(\beta ).$$

We note that the main algebraic structure is similar here and in (7.5)–(7.6) and (7.11)–(7.12). According to (A 4)–(A 6), the similarity transformed complex amplitudes are given by **x**′=[*x*′_1_ *x*′_2_]^T^=**S**^†^**x** and **v**′=[*v*′_1_ *v*′_2_]^T^=**S**^†^**v**, where **S**=[**e**_1_ **e**_2_] is the similarity transforming matrix—see (A 5).

By means of the similarity transformation, (7.3) may be simplified to

7.24 $$\begin{array}{rl} 2(P_{a,\mathrm{MAX}}-P_{a}) & ={\left({\text{v}}_{0}-\text{v}\right)}^{†}\text{D}({\text{v}}_{0}-\text{v})\\ & =\lambda_{1}|v_{10}^{\prime }(\beta )-v_{1}^{\prime }{|}^{2}+\lambda_{2}|v_{20}^{\prime }(\beta )-v_{2}^{\prime }{|}^{2}; \end{array}$$

see also (A 15). We note that (7.24) has an algebraic structure, as well as a geometrical interpretation, similar to that of (7.7).

For the particular case of a heaving axisymmetric body that contains an axisymmetric OWC, we have *J*^2^=*RG*, and thus, from (7.20), we see that *λ*_1_=*R*+*G* and *λ*_2_=0, which means that matrix **D**, in this case, is singular and of rank *r*_**D**_=1 [10, eqn 73]. In this case, there is only one term in the sum on the r.h.s. of (7.24), which simplifies to

7.25 $$2(P_{a,\mathrm{MAX}}-P_{a})=\sum_{i=1}^{2}\lambda_{i}|v_{i0}^{\prime }(\beta )-v_{i}^{\prime }{|}^{2}=(R+G)|v_{10}^{\prime }(\beta )-v_{1}^{\prime }{|}^{2},$$

which represents a circle in the complex *v*′_1_ plane. The centre of the circle is at *v*′_1_=*v*′_10_(*β*)=*x*′_1_(*β*)/(2*λ*_1_)=*x*′_1_(*β*)/(2*R*+2*G*), and the radius is $\sqrt{2(P_{a,\mathrm{MAX}}-P)/(R+G)}$. While figure 3 may serve to illustrate (7.24), figure 1 is more relevant as an illustration of (7.25). Because of the singularity of the radiation damping matrix, the similarity-transformed variable *v*′_2_ is irrelevant, and may have any arbitrary value, without influencing the absorbed power. The physical reason for the singularity is that both modes, the heaving-body mode and the OWC mode can radiate only isotropic outgoing waves. To realize maximum absorbed wave power, the optimum isotropically radiated wave may be realized by any optimum combined wave radiation from the axisymmetric OWC and the heaving axisymmetric body. The transformed oscillation *v*′_2_ corresponds to a situation where the heave mode and the OWC mode cancel each other's radiated waves in the far-field region.

## 8. Discussion

In this section, we first compare two versions of the so-called ‘fundamental theorem of wave power’ [17]. We shall discuss, secondly, the direction-averaged maximum absorbed wave power for an array of WEC units, and also, thirdly, the physical interpretation of the absorbed-wave-power surfaces. Finally, we shall comment on a disputed formula applied to the optimum performance of the Evans Cylinder.

### 8.1 The ‘fundamental theorem of wave power’

In this paper, by considering the physical process of wave-power absorption at the wetted surface of an oscillating immersed body, and, more generally, at a WEC array's wave-interacting surfaces, we derived, in §§3 and 6.2, respectively, an LPV version of the ‘fundamental theorem of wave power’, equation (3.11) : *P*_a_=*AE*(*β*)+A*E*(*β*)−|*U*|^2^. Moreover, we presented, in §4, a GPV version, equation (4.5): *P*_a_=*I*(*β*)+I*(*β*)−|*G*|^2^, where *I*(*β*)+I*(*β*) is the wave-power input through an envisaged surface enclosing all WEC units, and |*G*|^2^ is the outgoing wave power through the same envisaged surface, which, for mathematical convenience, is chosen in the far-field region of the generated waves. In §§5 and 6.1 we introduced a mixed, or hybrid, GLPV version, where the LPV parameters |*U*|^2^ and *E*(*β*), by means of (4.10), (5.2) and (6.5), are expressed in terms of global far-field quantities.

For these versions of the ‘fundamental theorem of wave power’, the r.h.s. has three terms, the sum of two complex conjugate terms minus a real, non-negative, term. The third term of the LPV version—including the GLPV version—contrary to the GPV version, is quadratically dependent on the oscillation amplitudes, but independent of the incident wave amplitude, while the first and second terms are linear in both kinds of amplitudes. For the LPV version, the first two terms, the excitation power, *P*_e_=*AE*(*β*)+A*E*(*β*), represent the gross power input from the incident wave, while the third term, *P*_r_=|*U*|^2^, is the necessary, outward-propagating, radiated power.

With the LPV/GLPV and GPV versions, the third terms |*U*|^2^ and |*G*|^2^, which represent energy associated with the radiated waves and the outgoing waves, as given by (4.10) and (4.6), respectively, should be considered as a necessity rather than a power loss. In order to absorb wave energy, it is necessary, firstly, to have wave-diffracting WEC units immersed in the sea, and, secondly, to let the WEC units oscillate and thus produce radiated waves, which interfere destructively with the incident wave. The WEC units need to oscillate in order to receive wave energy.

Before comparing the LPV/GLPV and GPV versions applied to a point absorber, let us consider a two-dimensional 100% absorbing WEC unit, such as an optimally run Evans Cylinder [2] or a hinged oscillating flap in the down-wave end of a wave channel, a flap that we may consider as an ideal nodding-duck device [23]. For these examples, the LPV equation (3.15) shows that the optimum values of the excitation power *P*_e_ and the radiated power *P*_r_ correspond to 200% and 100%, respectively. For the GPV version, which does not discriminate between radiated waves and diffracted waves, the optimum values of the input power, *P*_i_=*I*(*β*)+I*(*β*) and the outgoing power *P*_g_=|*G*|^2^ correspond to 100% and 0%, respectively. (Note that for a real nodding-duck WEC that absorbs less than 100%, the optimum outgoing power is not zero, and the optimum input power is larger than the maximum absorbed power.) As we shall see below, the two versions show a less drastic difference when applied to a point absorber.

In a case where the WEC array is not oscillating, there is no absorbed wave power, *P*_a_|_**v**=**0**_=0, and also no radiated power, *P*_r_|_**v**=**0**_=0. Then it follows from (3.11) that *P*_e_|_**v**=**0**_=0, and, moreover, from (4.5) that *P*_i_|_**v**=**0**_=*P*_g_|_**v**=**0**_≡*P*_d_, where *P*_d_ is the outgoing power associated with the diffracted wave alone. From (4.8), we may note that *P*_i_|_**v**=**0**_=*P*_d_=*ρv*_p_*v*_g_Re{*H*_d_(*β*)A*}≥0.

In cases of rather weak diffraction, as with a wave-power-absorbing very small point absorber, *P*_d_ may be negligibly small. We may note that, if *H*_d_(*θ*) is small for all *θ* (including *θ*=*β*), then the r.h.s. of (4.9) is small of second order. Thus, in cases of very weak diffraction, *H*_d_(*β*) is, approximately, purely imaginary, if we choose *A* to be real. This matter has been discussed in more detail by Farley [15]. By comparing (4.7) with (4.11) and (4.6) with (4.10), we observe that, for cases where the diffracted wave is negligible compared to the radiated wave, *I*(*β*)≈*E*(*β*)*A* and |*G*|^2^≈|*U*|^2^. Thus, for such cases, there is no great difference between corresponding terms of the LPV/GLPV and GPV versions of the ‘fundamental theorem of wave power’.

In §3, oscillations, wave forces, power and energy were quantitatively discussed in the time domain, but elsewhere, in this paper, only in the frequency domain. In the case of non-sinusoidal waves, it may be desirable to carry out analyses in the time domain. In this situation, a time-domain type of the ‘fundamental theorem of wave power’ may be desirable. This type should correspond to an inverse Fourier transform of the LPV version derived in §6.2—or §3 for the one-mode case. It should neither be the GPV version nor the GLPV version, which are derived and discussed in §§4, 5 and 6.1. These versions cannot represent the instantaneous power absorbed by the WEC, but only the time-average power. With a time-domain analysis, also the reactive-power terms—see §3 and §6.2—need to be taken into account.

### 8.2 Direction-averaged maximum absorbed wave power

For the case of only one immersed WEC unit oscillating in a single mode *i*=1, we have, in agreement with (4.3) and (5.3), that *H*_r0_(*θ*)=*h*_1_(*θ*)*v*_10_(*β*), and that ${\bar{H}}_{r0}(\beta +\pi )=h_{1}(\beta +\pi )v_{10}^{*}(\beta )$. Then the optimum gain function (6.11) simplifies to

8.1 $$G_{g0}(\beta )=\frac{2\pi |h_{1}(\beta +\pi ){|}^{2}}{\int_{0}^{2\pi }|h_{1}(\theta ){|}^{2}\;d\theta }.$$

Since the Kochin function coefficient *h*_1_(*θ*) is a function of geometry and mode of motion, this means that the optimum gain function *G*_g0_ for this case depends on geometry and mode of motion only, and not on the WEC velocity. However, to maximize power absorption, the WEC unit needs to move with optimum amplitude and phase. For an isotropically radiating system, such as a heaving axisymmetric body, the optimum gain function is *G*_g0_=1 and independent of the wave-incident angle *β*. Then, (6.10) gives the maximum absorption width *d*_a,MAX_≡*P*_a,MAX_/*J*_w_=*G*_g0_/*k*=1/*k*, a well-known result since the mid-1970s.

From (8.1), we find that the direction-averaged optimum gain function is

8.2 $$G_{g0,\mathrm{average}}=\frac{1}{2\pi }\int_{0}^{2\pi }G_{g0}(\beta )\;d\beta =1,$$

as averaged over all directions of wave incidence, a result reported by Fitzgerald & Thomas [31]. However, in some singular cases, we may find that *G*_g0,average_=0. For instance, any axisymmetric body oscillating only in the yaw mode can, in an ideal fluid, neither radiate nor absorb wave energy, for any frequency. Then *H*_r_(*θ*)≡0. For a floating semi-submersible platform, as well as for a floating bottle-shaped axisymmetric body that has a relatively small water-plane area, the heave excitation force vanishes at a certain frequency [32, p. 77]. Hence, according to the Haskind relation (5.1), *h*_1_(*β*+*π*)=0, and then *G*_g0_(*β*)=0 at this particular frequency.

For a general WEC array oscillating in *N* modes, with *N*≥2, it is not convenient to apply (6.11) to determine *G*_g0,average_. By means of another mathematical procedure, involving similarity transformation, as applied in appendix A, it is found that *G*_g0,average_, in general, equals the rank *r*_**D**_ of the radiation damping matrix **D**—see (A 17). In cases where this matrix is non-singular, the rank of the matrix equals its dimensionality *N*. Thus, in general, the direction-averaged value of the optimum gain function is equal to an integer in the interval 0≤*G*_g0,average_≤*N*. For instance, an immersed body may oscillate in *N*=6 different modes. However, if the body has a vertical axis of symmetry, then *G*_g0,average_=3 in general, or less in exceptional cases [1, §10]. These results extend the findings of Wolgamot *et al.* [18], who found the result *G*_g0_(*β*)=*N* for cases where the, general, hermitian radiation-damping matrix **D** specializes to a, non-singular, real radiation-resistance matrix.

We considered in §7.2 an array consisting of two heaving point absorbers, and we derived formula (7.17) for the maximum absorbed power. To find the direction-averaged maximum absorbed power, we need to integrate cos⁡{kdcos⁡β} from *β*=0 to *β*=2*π*. Since this integral equals *J*_0_(*kd*) (e.g. [33, formula 9.1.18, p. 360]) we see, from (7.17) and the first equation of (7.14), that the direction-average of the absorbed power is |*F*_0_|^2^/(4*R*_0_)=2*J*_w_/*k*. This result was, according to (A 1) or (A 17), to be expected for this non-singular system of two heaving bodies.

### 8.3 Absorbed-wave-power surfaces

The absorbed wave power relative to its maximum may be expressed as two equally general functions of the WEC oscillation amplitudes relative to the optimum amplitudes. The first of these expressions is given in (3.17), which may be illustrated as an axisymmetric paraboloid on the complex collective-amplitude *U* plane (figure 1). The second expression is given in (6.24), which may be thought of as a ‘paraboloid’ in the complex *N*-dimensional **v** space, $C^{N}$ (see figure 3 for an example with *N*=2).

In popular terms, it might be useful to think of this ‘paraboloid’, for *P*_a_>0, as a single ‘mountain island’ in a $C^{N}$ ‘world’, where there is otherwise, for *P*_a_<0, only an infinite ‘ocean’ (cf. figures 1 and 3). The top of the absorbed-power ‘mountain’ corresponds to optimum, and the ‘shore’ of the ‘island’ to *P*_a_ = 0. This ‘mountain’ top can be reached only if no technical or practical constraint prevents the required complex amplitudes *v*_*i*0_ from being realized for all *i*=1,2,3,…,*N*.

For practical reasons, it may not be possible to realize the optimum condition **v**=**v**_0_. Note that all components of the excitation vector **x**(*β*), and hence also of the optimum oscillation amplitude vector **v**_0_(*β*), are proportional to the complex amplitude *A* of the wave elevation of the undisturbed incident wave, and that *P*_a,MAX_ is proportional to A*A=|*A*|^2^. As oscillation amplitudes cannot exceed their design-specified limits, it will not be possible to realize the described optimum situation, if the amplitude of the incident wave exceeds a certain critical value. With such constraints, or for other technical reasons preventing realization of the optimum condition (6.23), the practical, constrained-case, maximum absorbed power *P*_a,max_ will be less than the ideal *P*_a,MAX_. (In such a case, it will not be practically possible to ‘climb’ to the ‘top’ of the above envisaged ‘mountain island’.)

### 8.4 A disputed 1979 formula

For the optimum case, the maximum absorption width may be expressed as

8.3 $$d_{a,\mathrm{MAX}}(\beta )=\frac{2\pi |{\bar{H}}_{r0}(\beta +\pi ){|}^{2}}{k\int_{0}^{2\pi }|H_{r0}(\theta ){|}^{2}\;d\theta },$$

according to (6.9)–(6.11). The single-mode version of this formula was derived by Newman [25] and, independently, by Evans [8]. For the case of more than one oscillation mode, we have found it necessary to introduce the adjoint Kochin function ${\bar{H}}_{r}$ in the numerator (see (5.3) and (6.4)).

It should be emphasized that formula (8.3) applies only to the optimum case, as it is based on the fact that the optimum radiated power is equal to the maximum absorbed wave power. Neither any wave force nor the incident wave amplitude is explicitly present in (8.3). However, as each component *v*_*i*0_ of the optimum oscillation vector is proportional to the incident wave amplitude, the numerator, as well as the denominator, of fraction (8.3) is proportional to the square of the incident wave amplitude.

The controversy [15,16]—concerning the numerator in formula (8.3)—is mainly related to the Evans Cylinder, which we, in the last three paragraphs of §7.1, discussed in some detail. Let this cylinder be aligned perpendicular to the incident wave direction. At optimum oscillation, this submerged horizontal cylinder absorbs all incident wave energy. Then the optimum radiated wave has to propagate only down-wave in order to cancel the transmitted wave, as there is no reflected wave to cancel up-wave. In agreement with this physical observation, it is reassuring to observe that we, according to (7.9), which is valid for the Evans Cylinder, may replace $|{\bar{H}}_{r0}(\beta +\pi )|$ by |*H*_r0_(*β*)| in the numerator of (8.3). Such a replacement seems to resolve the controversy, because *H*_r0_(*β*)=*h*_1_(*β*)*u*_10_+*h*_2_(*β*)*u*_20_ is the Kochin function for the down-wave radiated wave when optimum wave-power absorption is actually taking place, that is, when the rotating Evans Cylinder's surge and heave modes' complex velocity amplitudes have their optimum values, *u*_10_ and *u*_20_, respectively. In comparison, ${\bar{H}}_{r0}(\beta +\pi )=h_{1}(\beta +\pi )u_{10}^{*}+h_{2}(\beta +\pi )u_{20}^{*}$ is the adjoint Kochin function, which corresponds to a wave that is radiated in the opposite direction if the rotating Evans Cylinder is oscillating with opposite sense of rotation. Observe that to replace *u*_*i*0_ by $u_{i0}^{*}$ corresponds to time reversal, since (e^i*ωt*^)*=*e*^i*ω*(−*t*)^.

It seems that we have to include the relation (8.3) among the reciprocity relations between physical quantities about which Newman [1, §7] expressed that they ‘are not physically related to each other in any obvious manner’.

## 9. Conclusion

After the petroleum crisis in 1973, the basic theory for primary wave-energy conversion was developed during the mid- and late 1970s and the early 1980s. Different versions—the LPV version (cf. §§3 and 6.2), the GLPV version (cf. §§5 and 6.1) and the GPV version (cf. §4)—of the so-called ‘fundamental theorem of wave power’ have given rise to some controversy even during recent years. Comparative discussion of these different versions has been presented in §8.1. The GLPV version, in particular, is mathematically convenient when proving some useful reciprocity relations, as applied, for instance, by Newman [1]. It is, however, difficult to give a physical interpretation of some of these relations and of the GLPV version. This may be the cause of recent controversy concerning the GLPV version. All of these versions provide, however, the correct value of the *time-average* absorbed wave power. Hopefully, the discussion in §8.4 helps to do away with some of this controversy.

The LPV, the GPV and the GLPV versions express, respectively, the absorbed wave power *P*_a_ in terms of the WEC units' complex oscillation amplitudes, in terms of the outgoing (diffracted plus radiated) wave, and in terms of the radiated wave alone. For mathematical convenience, the outgoing and radiated waves in the far-field region are considered, explicitly.

For a general WEC array consisting of oscillating immersed bodies and OWCs, we have found it convenient to introduce complex collective parameters, the collective oscillation amplitude *U* and the collective excitation-power coefficient *E*(*β*) (see (3.10) and (6.20)). Then it is, even for a WEC array, a rather simple algebraic exercise to derive expressions for the maximum absorbed wave power *P*_a,MAX_ and the corresponding optimum values *U*_0_ and *E*_0_(*β*) (see (3.11)–(3.15)). Moreover, we may illustrate the dependence of the absorbed wave power *P*_a_ versus *U* as an axisymmetric paraboloid; cf. (3.17) and figure 1. For an *N*-mode WEC array, we may, in greater detail than figure 1, consider the real-valued *P*_a_ as represented by a paraboloid in an *N*-dimensional complex **v** space $C^{N}$. Cross sections of such a paraboloid are, as an example, illustrated in figure 3. Mathematically, the mentioned paraboloids are represented by rather simple mathematical expressions (3.17) and (6.24), which may be considered as alternative variants of the LPV version of the ‘fundamental theorem of wave power’.

In contrast to the GPV version and the GLPV version, only the LPV version is applicable for the purpose of quantifying the *instantaneous* absorbed wave power. Then it is necessary to take also the reactive power into account. When deriving the LPV version (cf. §§3 and 6.2), we also discussed the reactive power that is associated with wave-power absorption. In appendix B, we have derived expressions that relate the ‘added-mass’ matrix, as well as a couple of additional reactive radiation-parameter matrices, to the difference between kinetic energy and potential energy in the water surrounding the immersed oscillating WEC array. To the best of the authors' knowledge, some of these derived relations are new results (e.g. (B 25), (B 27) and (B 28)). In appendix B.5, we have also derived new relations concerning reactive power associated with the incident wave.

In appendix A, we applied similarity transformation of the radiation damping matrix to derive a formula for the direction-average maximum absorbed wave power *P*_a,*MAX*,average_. Correspondingly, as discussed in §8.2, we found that the direction-average value *G*_g0,average_ of the optimum gain function *G*_g0_(*β*)—defined by (6.11)—equals an integer in the interval 0≤*G*_g0,average_≤*N*, where *N* is the WEC array's number of modes of oscillation (number of degrees of motion). Only when the radiation damping matrix is non-singular, we have *G*_g0,average_=*N*, as derived by Wolgamot *et al.* [18, eqn 21] for an *N*-mode WEC array consisting of oscillating bodies only. Thus, our result is an extension of theirs, to WEC arrays that may contain OWCs and also may have a singular radiation damping matrix. In general, *G*_g0,average_ equals the rank of this *N*×*N* matrix.

## Appendix A. Direction-average maximum absorbable wave power

Consider wave-energy absorption by an array of immersed oscillating rigid bodies and OWCs. The number of wave-interacting oscillators is *N*=*N*_u_+*N*_p_, where *N*_p_ is the number of OWCs and *N*_u_ is the number of used body modes, which may be up to six times the number of bodies.

Observing, e.g. from (6.10) and (6.22), that the maximum absorbed power *P*_a,MAX_=*P*_a,MAX_(*β*), our present aim is to prove the following conjecture [34, p. 17]:

A 1 $$P_{a,\mathrm{MAX},\mathrm{average}}\equiv \frac{1}{2\pi }\int_{-\pi }^{\pi }P_{a,\mathrm{MAX}}(\beta )\;d\beta =\frac{r_{\text{D}}\;J_{w}}{k},$$

where *r*_**D**_ is the rank of the radiation damping matrix **D**, as given in (6.15). For cases when matrix **D** is non-singular, its rank equals its dimensionality, *r*_**D**_=*N*. For an array without OWCs and a non-singular radiation resistance *N*_u_×*N*_u_ matrix **R**, which is real, symmetric and positive definite and has a rank of *r*_**R**_=*N*_u_, Wolgamot *et al.* [18, eqn 21] derived a result in agreement with our conjecture (A 1).

However, in many cases of interest, the radiation matrix is singular, that is, its determinant vanishes, |**D**|=0. In such cases, it is possible to find a linear combination of two different oscillation modes, in such a way that the resulting far-field radiated wave vanishes. This is possible, for instance, with certain linear combinations of surge and pitch modes for an axisymmetric body [35, p. 175]. A two-dimensional example, a symmetric body, which has *N*_u_=3 modes of motion (surge, heave and pitch), was discussed by Newman [1], who found that two modes (heave and surge or heave and pitch) are sufficient to absorb 100% of the incident wave energy; thus a third mode is not needed. Newman also studied optimum oscillation for maximum wave power absorbed by one body which has a vertical symmetry axis and oscillates in the three modes surge, heave and pitch—an example where either surge or pitch was found not necessary. Including the three remaining modes (sway, roll and yaw) for this axisymmetric body, we find that its radiation resistance matrix **R** has a dimensionality *N*_u_=6, while its rank is only *r*_**R**_=3 (cf. [35, Subsection 6.4.2 & Problems 5.13 & 6.3]). Evidently, the conjecture (A 1) holds for the case of immersed oscillating bodies that are symmetric about a vertical axis [34, p. 17].

**A.1. Similarity transformation**

To derive a result in agreement with our conjecture (A 1), Wolgamot *et al.* [18] applied Cholesky decomposition of the real symmetric radiation resistance matrix **R**, provided this matrix is positive definite and thus non-singular. It would be straightforward to apply the same method for our complex hermitian radiation damping matrix **D** in cases where this matrix is non-singular and thus positive definite. However, we wish to include the general case when matrix **D** may be singular and thus need to have at least one of its eigenvalues equal to zero. In this case, Cholesky decomposition is less convenient.

Instead we shall apply similarity transformation (e.g. [36, ch. V]) to prove our conjecture (A 1). Then our first step is to determine the eigenvalues *λ*_*i*_ and the corresponding, normalized and mutually orthogonal, eigenvectors **e**_*i*_ for *i*=1,2,3,…,*N*−1,*N*. They have to satisfy the following system of linear homogeneous equations:

A 2 $$\text{D}{\text{e}}_{i}=\lambda_{i}{\text{e}}_{i}.$$

Because matrix **D** is hermitian, all its eigenvalues are real, and since it is positive semidefinite, they are all non-negative. They are the *N* solutions of the *N*-degree equation |**D**−*λ***I**|=0, where **I**=diag(1,1,1,…,1) is the *N*×*N* identity matrix, for which all diagonal entries equal 1, while all other entries equal 0. Further, |**D**−*λ***I**| denotes the determinant of matrix (**D**−*λ***I**). We may wish to arrange the *N* eigenvalues in descending order, that is,

A 3 $$\lambda_{1}\geq \lambda_{2}\geq \lambda_{3}\geq \cdots \geq \lambda_{N}\geq 0.$$

Note that if **e**_*i*_ is a possible solution when *λ*=*λ*_*i*_, then also *C*_*i*_**e**_*i*_ is a solution, where *C*_*i*_ is an arbitrary complex scalar. It is convenient to normalize the eigenvectors **e**_*i*_ in such a way that the condition

A 4 $${\text{e}}_{i}^{†}{\text{e}}_{j}=\delta_{ij}=\left\{\begin{array}{ll} 1 & \text{for }i=j\\ 0 & \text{for }i\neq j \end{array}\right.$$

applies. With this choice we may consider this particular set of eigenvectors to be a complete set of, mutually orthogonal, unit vectors in our *N*-dimensional complex space.

We now define the *N*×*N* matrix

A 5 $$\text{S}=[{\text{e}}_{1}\;{\text{e}}_{2}\;{\text{e}}_{3}\;\cdots \;{\text{e}}_{N}].$$

It then follows from (A 4) that **S**^†^**S**=**I**. Hence, **S**^−1^=**S**^†^. We are now ready to carry out a similarity transformation of vectors **v** and **x**, defined in (6.1). Let

A 6 $${\text{v}}^{\prime }={\text{S}}^{-1}\text{v}={\text{S}}^{†}\text{v}\;\text{and}\;{\text{x}}^{\prime }={\text{S}}^{-1}\text{x}={\text{S}}^{†}\text{x},$$

and conversely, **v**=**S****v**′ and **x**=**S****x**′. Then, the collective excitation-power coefficient *E*(*β*) and the collective oscillation amplitude *U* defined in (6.20) may be expressed as *E*(*β*)=**v**^†^**x**/(4*A*)=**v**′^†^**x**′/(4*A*) and |*U*|^2^=**v**^†^**D****v**/2=**v**′^†^**D**′**v**′/2, where

A 7 $${\text{D}}^{\prime }={\text{S}}^{†}\text{D}\text{S}=\text{diag}(\lambda_{1},\lambda_{2},\ldots ,\lambda_{N}).$$

We have here made use of (A 2), (A 4) and (A 5) to obtain the last equality. Note that matrix **D**′ has the same eigenvectors and eigenvalues, and hence also the same rank, as matrix **D**. While **D** is complex and hermitian in the general case, the diagonal matrix **D**′ is real. Moreover, the complex collective parameters *E*(*β*) and *U* remain unchanged through the similarity transformation.

By means of a well-known reciprocity relation [10], we may express the radiation damping matrix **D** in terms of the wave excitation vector **x** as follows:

A 8 $$\text{D}=\frac{k}{16\pi J_{w}}\int_{-\pi }^{\pi }\text{x}(\beta ){\text{x}}^{†}(\beta )\;d\beta ,$$

where *k* is the angular repetency (wave number) and *J*_w_ is the wave power level (incident wave power transport per unit width of the wave front), defined in (2.2). Using (A 6)–(A 8), we obtain

A 9 $${\text{D}}^{\prime }={\text{S}}^{†}\text{D}\text{S}=\frac{k}{16\pi J_{w}}\int_{-\pi }^{\pi }{\text{S}}^{†}\text{x}(\beta ){\text{x}}^{†}(\beta )\text{S}\;d\beta =\frac{k}{16\pi J_{w}}\int_{-\pi }^{\pi }{\text{x}}^{\prime }(\beta ){\text{x}}^{\prime †}(\beta )\;d\beta .$$

Moreover, the optimum condition (6.23) may be reformulated as

A 10 $${\text{D}}^{\prime }{\text{v}}_{0}^{\prime }(\beta )=\frac{1}{2}{\text{x}}^{\prime }(\beta ),\;\text{that is,}\;\lambda_{i}v_{i0}^{\prime }(\beta )=\frac{1}{2}x_{i}^{\prime }(\beta )\;\text{for}\;i=1,2,\ldots ,N,$$

and the maximum absorbed power, as given by (6.22), may be rewritten as

A 11 $$P_{a,\mathrm{MAX}}(\beta )=\frac{1}{2}{{\text{v}}_{0}^{\prime }}^{†}(\beta ){\text{D}}^{\prime }{\text{v}}_{0}^{\prime }(\beta )=\frac{1}{2}\sum_{i=1}^{N}\lambda_{i}v_{i0}^{\prime *}(\beta )v_{i0}^{\prime }(\beta )=\frac{1}{2}\sum_{i=1}^{N}\lambda_{i}|v_{i0}^{\prime }(\beta ){|}^{2}.$$

In the general case, the *N*×*N* matrix **D** may be singular, and then of rank *r*_**D**_<*N*. For convenience, let its *N* eigenvalues be arranged to satisfy the following condition:

A 12 $$\lambda_{1}\geq \lambda_{2}\geq \cdots \geq \lambda_{r_{\text{D}}}>\lambda_{r_{\text{D}}+1}=\lambda_{r_{\text{D}}+2}=\cdots =\lambda_{N}=0,$$

where the integer *r*_**D**_ satisfies 1≤*r*_**D**_≤*N*. The matrix is non-singular if *r*_**D**_=*N*. From (A 7) and (A 9), we may now conclude that the only non-zero matrix-element entries of our transformed radiation damping matrix **D**′ are the following *r*_**D**_ diagonal entries:

A 13 $$D_{ii}^{\prime }=\lambda_{i}=\frac{k}{16\pi J_{w}}\int_{-\pi }^{\pi }|x_{i}^{\prime }(\beta ){|}^{2}\;d\beta \;\text{for }i=1,2,\ldots ,r_{\text{D}}.$$

Note from (A 10) that at optimum, if *λ*_*i*_≠0, the oscillation component *v*′_*i*0_(*β*) has to be in phase with the excitation component *x*′_*i*_(*β*). In general, because matrix **D** may have non-zero off-diagonal entries, unless *N*=1, such a simple optimum-phase relationship does not apply between *v*_*j*0_(*β*) and *x*_*j*_(*β*), components of vectors **v**_0_(*β*) and **x**(*β*), where *j*=1,2,…,*N*. The smallest positive eigenvalue is *λ*_*r*_**D**__. Observe that if it is very small, then there is a risk that the required optimum amplitude |*v*′_0__*r*_**D**__(*β*)| may be impractically large! Note that for *r*_**D**_<*i*≤*N*, we have no particular optimum requirement for *v*′_*i*0_(*β*). Then the optimum value of **v**_0_(*β*) is ambiguous, although the maximum absorbed power is unambiguous, as is made clear by the following equation (A 14).

Using (A 10) and (A 12), the maximum absorbed power (A 11) can now be written as

A 14 $$P_{a,\mathrm{MAX}}(\beta )=\frac{1}{2}\sum_{i=1}^{N}\lambda_{i}|v_{i0}^{\prime }(\beta ){|}^{2}=\frac{1}{2}\sum_{i=1}^{r_{\text{D}}}\lambda_{i}|v_{i0}^{\prime }(\beta ){|}^{2}=\frac{1}{8}\sum_{i=1}^{r_{\text{D}}}\frac{|x_{i}^{\prime }(\beta ){|}^{2}}{\lambda_{i}}.$$

This result represents a generalization of a previous expression [37, eqn 135] for the maximum wave power absorbed by an array consisting of oscillating rigid bodies to a more general situation when the array contains also OWCs. Also, by using (A 6), (A 7) and (A 12), equation (6.24) may be expressed as

A 15 $$P_{a,\mathrm{MAX}}(\beta )-P_{a}=\frac{1}{2}{\left\{{\text{v}}^{\prime }-{\text{v}}_{0}^{\prime }(\beta )\right\}}^{†}{\text{D}}^{\prime }\{{\text{v}}^{\prime }-{\text{v}}_{0}^{\prime }(\beta )\}=\frac{1}{2}\sum_{i=1}^{r_{\text{D}}}\lambda_{i}|v^{\prime }-v_{i0}^{\prime }(\beta ){|}^{2},$$

which, for any fixed positive l.h.s. value, represents an ‘ellipsoid’ in the complex *r*_**D**_-dimensional **v** space, $C^{r_{\text{D}}}$.

In agreement with (A 14), the maximum absorbed wave power averaged over all angles of wave incidence may be written as

A 16 $$P_{a,\mathrm{MAX},\mathrm{average}}=\frac{1}{2\pi }\int_{-\pi }^{\pi }P_{a,\mathrm{MAX}}(\beta )\;d\beta =\frac{1}{16\pi }\sum_{i=1}^{r_{\text{D}}}\frac{1}{\lambda_{i}}\int_{-\pi }^{\pi }|x_{i}^{\prime }(\beta ){|}^{2}\;d\beta .$$

Using (A 13) to eliminate the integral, we finally obtain

A 17 $$P_{a,\mathrm{MAX},\mathrm{average}}=\frac{1}{2\pi }\int_{-\pi }^{\pi }P_{a,\mathrm{MAX}}(\beta )\;d\beta =\sum_{i=1}^{r_{\text{D}}}\frac{J_{w}}{k}=\frac{r_{\text{D}}J_{w}}{k},$$

in agreement with our conjecture (A 1), which we have now proved to be correct!

**A.2. Two-body example**

As a simple example of similarity transformation, consider a system of two equal axisymmetric bodies with their vertical symmetry axes located at (*x*,*y*)=(∓*d*/2,0). We shall assume that they are oscillating in the heave mode only. With this assumption, the excitation-force vector is of the form **x**=**F**_e_=[*F*_e,1_ *F*_e,2_]^T^. Further, the radiation resistance matrix may be written as the 2×2 matrix

A 18 $$\text{R}=\left[\begin{array}{cc} R_{d} & R_{c}\\ R_{c} & R_{d} \end{array}\right].$$

Note that the diagonal entry *R*_d_ is positive, while the off-diagonal entry *R*_c_ may be positive or negative, depending on the distance *d* between the two bodies. Since waves radiated from the two distinct bodies cannot cancel each other in all directions in the far-field region—which may be proved, by summing two asymptotic expressions (e.g. [35, formulae 4.266 and 4.270])—the matrix **R** is non-singular, and hence $R_{c}^{2}<R_{d}^{2}$. (We assume that the body does not have such a peculiar shape that we may find *R*_d_ to vanish for some particular frequency.)

The eigenvalues *λ*=*λ*_1,2_ satisfy the algebraic equation $0=|\text{R}-\lambda \text{I}|={\left(R_{d}-\lambda \right)}^{2}-R_{c}^{2}=(\lambda -R_{d}-R_{c})(\lambda -R_{d}+R_{c})\equiv (\lambda -\lambda_{1})(\lambda -\lambda_{2})$; thus

A 19 $$\lambda_{1}=R_{d}+R_{c}>0\;\text{and}\;\lambda_{2}=R_{d}-R_{c}>0.$$

It is easy to show that, for this case, the corresponding eigenvectors **e**_1_ and **e**_2_, as defined by (A 2), and normalized in agreement with (A 4), are given by

A 20 $$[{\text{e}}_{1}\;{\text{e}}_{2}]=\frac{1}{\sqrt{2}}\left[\begin{array}{cc} 1 & 1\\ 1 & -1 \end{array}\right]=\text{S}={\text{S}}^{T}={\text{S}}^{†}={\text{S}}^{-1},$$

where we have also used the definition (A 5). In accordance with (6.1) and (A 6), the excitation-force and the optimum oscillation-velocity vectors transform as

A 21 $${\text{F}}_{e}^{\prime }=\left[\begin{array}{c} F_{e,1}^{\prime }\\ F_{e,2}^{\prime } \end{array}\right]={\text{S}}^{-1}{\text{F}}_{e}=\frac{1}{\sqrt{2}}\left[\begin{array}{cc} 1 & 1\\ 1 & -1 \end{array}\right]\left[\begin{array}{c} F_{e,1}\\ F_{e,2} \end{array}\right]=\frac{1}{\sqrt{2}}\left[\begin{array}{c} F_{e,1}+F_{e,2}\\ F_{e,1}-F_{e,2} \end{array}\right]$$

and

A 22 $${\text{u}}_{0}^{\prime }=\left[\begin{array}{c} u_{10}^{\prime }\\ u_{20}^{\prime } \end{array}\right]={\text{S}}^{-1}{\text{u}}_{0}=\frac{1}{\sqrt{2}}\left[\begin{array}{cc} 1 & 1\\ 1 & -1 \end{array}\right]\left[\begin{array}{c} u_{10}\\ u_{20} \end{array}\right]=\frac{1}{\sqrt{2}}\left[\begin{array}{c} u_{10}+u_{20}\\ u_{10}-u_{20} \end{array}\right],$$

respectively. For this example, where the 2×2 matrix **R** is non-singular, (A 14)–(A 17) are applicable with *r*_**D**_=*N*=*N*_u_=2. For this case, (A 14) specializes to

A 23 $$P_{a,\mathrm{MAX}}(\beta )=\frac{|F_{e,1}^{\prime }(\beta ){|}^{2}}{8\lambda_{1}}+\frac{|F_{e,2}^{\prime }(\beta ){|}^{2}}{8\lambda_{2}}=\frac{|F_{e,1}(\beta )+F_{e,2}(\beta ){|}^{2}}{16(R_{d}+R_{c})}+\frac{|F_{e,1}(\beta )-F_{e,2}(\beta ){|}^{2}}{16(R_{d}-R_{c})},$$

where we, in the last step, have made use of (A 19) and (A 21).

## Appendix B. Difference between kinetic energy and potential energy in the near-field region

It is well known that, for a propagating plane wave, the time-average energy stored per unit horizontal area *E*=*E*_k_+*E*_p_ is divided into equal amounts of kinetic energy and potential energy. Thus, *E*_k_=*E*_p_=*E*/2, that is, *E*_k_−*E*_p_=0. As will be evident through a derivation below, the same is true for a radiated, outwards propagating, circular wave in the far-field region. However, generally, in the near-field region of a group of oscillating immersed bodies, *E*_k_−*E*_p_≠0. For a case with no incident wave, this difference is related to the added-mass matrix **m** for immersed radiating bodies performing forced oscillations, through the relationship [19,38]

B 1 $$T-V=\frac{1}{4}{\text{u}}^{†}\text{m}\text{u}=\frac{1}{4\omega }{\text{u}}^{†}\text{X}\text{u}.$$

Here **u**=[*u*_1_ *u*_2_ *u*_3_ ⋯ *u*_*N*_u__]^T^ is the vector of complex velocity amplitudes of the *N*_u_ used body modes. The l.h.s. of (B 1) denotes the total near-field value of the difference between kinetic and potential energy, namely,

B 2 $$T-V=\lim_{kr\to \infty }(W_{k}-W_{p})=\lim_{kr\to \infty }\iint_{S_{0}}(E_{k}-E_{p})\;dS,$$

where *S*_0_ is the air–water interface. We have, in (B 1), also introduced the radiation-reactance matrix **X**=*ω***m**. (This is a less confusing terminology than ‘added mass’ for cases where *V* >*T*.)

Our aim is to generalize (B 1) to a situation where the array contains *N*_p_ OWC oscillating modes in addition to the rigid bodies, as indicated in figure 2. Let the column vector **p**=[*p*_1_ *p*_2_ *p*_3_ ⋯ *p*_*N*_p__]^T^ denote the oscillating state of the *N*_p_ OWCs, where *p*_*k*_ is the complex amplitude of the dynamic air pressure of OWC *k*. For such a more general case, the integral on the r.h.s. of (B 2) needs to be replaced by integrals derived below. We shall find, as a result, that a generalized version of (B 1) has to contain additional terms on the r.h.s., including terms associated with the incident wave.

**B.1. Preparatory basic equations**

In accordance with linear theory for an ideal fluid, the complex amplitudes *p*=−i*ωρϕ* of the hydrodynamic pressure and ***v***=∇*ϕ* of the fluid (water) velocity are derivable from the velocity potential *ϕ*, which, everywhere in the ideal fluid, has to obey the Laplace equation ∇^2^*ϕ*=0. Moreover, *ϕ* has to satisfy certain boundary conditions as shown below. The complex amplitude of the water-surface elevation *η* on *S*_0_, as well as *η*_*k*_ on *S*_*p*,*k*_=*S*_*k*_ (figure 2) is given by

B 3 $$\eta ={\left.-\frac{i\omega }{g}\phi \right|}_{S_{0}}\;\text{as well as}\;\eta_{k}={\left.-\frac{i\omega }{g}\phi \right|}_{S_{k}}-\frac{1}{\rho g}p_{k},$$

respectively. Here the water–air interfaces, outside the OWC structures and inside the *k*th OWC structure, are denoted by *S*_0_ and *S*_*p*,*k*_, respectively. (It is assumed that the ambient air pressure is static.) Moreover, *ρ* is the mass density of water, *g* is the acceleration of gravity and *ω* is the angular frequency of the considered waves and oscillations. The potential energy, associated with the wave, results from water being lifted against gravity, and inside the *k*th OWC also against the dynamic air pressure *p*_*k*_. On *S*_0_ and *S*_*p*,*k*_, the time-average of the potential energy, per unit horizontal surface, is

B 4 $$E_{p}{|}_{S_{0}}=\frac{\rho g|\eta {|}^{2}}{4}\;\text{and}\;E_{p}{|}_{S_{p,k}}=\frac{\rho g|\eta_{k}{|}^{2}+p_{k}\eta_{k}^{*}+p_{k}^{*}\eta_{k}}{4},$$

respectively.

For convenience, we decompose the velocity potential *ϕ* into three terms, *ϕ*_0_, *ϕ*_*d*_ and *ϕ*_*r*_, corresponding to the incident, the diffracted and the radiated waves:

B 5 $$\phi =\phi_{0}+\phi_{d}+\phi_{r}=\phi_{s}+\phi_{r}=\phi_{s}+\varphi_{u}^{T}\text{u}+\varphi_{p}^{T}\text{p}=\phi_{s}+\sum_{i=1}^{N_{u}}\varphi_{u,i}\;u_{i}+\sum_{k=1}^{N_{p}}\varphi_{p,k}\;p_{k},$$

where *ϕ*_*s*_=*ϕ*_0_+*ϕ*_d_ is the scattered potential. Further, we have introduced column vectors ***φ***_*u*_=[*φ*_*u*,1_ *φ*_*u*,2_ ⋯ *φ*_*u*,*N*_u__]^T^ and ***φ***_*p*_=[*φ*_*p*,1_ *φ*_*p*,2_ ⋯ *φ*_*p*,*N*_p__]^T^, which are composed of space-dependent, complex, proportionality coefficients *φ*_*u*,*i*_ and *φ*_*p*,*k*_, that quantify how the radiated potential *ϕ*_*r*_ is linearly related to *u*_*i*_ and *p*_*k*_, components of the oscillation-state column vectors **u** and **p**, respectively.

It is required that every term in (B 5) satisfies the Laplace equation. Furthermore, it is required that every term in (B 5), except *ϕ*_0_—and, consequently, also except *ϕ*_*s*_ and *ϕ*—satisfies the radiation condition of outgoing waves at infinity, that is

B 6 $$\lim_{kr\to \infty }\frac{\partial [\phi_{d}\;\phi_{r}\;\varphi_{u}^{T}\;\varphi_{p}^{T}]}{\partial r}=-ik\lim_{kr\to \infty }[\phi_{d}\;\phi_{r}\;\varphi_{u}^{T}\;\varphi_{p}^{T}],$$

where *r* is the radius of the envisaged control cylinder $S_{\infty }$ (figure 2). It may be remarked that, for large values of *kr*, all these complex amplitudes, *ϕ*_d_, *ϕ*_*r*_, ***φ***_*u*_ and ***φ***_*p*_, diminish asymptotically as $1/\sqrt{kr}$ when kr→∞, and their directional variation may, quantitatively, be described by certain complex factors *s*_d_(*θ*), *s*_r_(*θ*), **s**_*u*_(*θ*) and **s**_*p*_(*θ*), respectively.

Furthermore, it is required that the components of the velocity potential in (B 5) satisfy certain boundary conditions. The following homogeneous boundary conditions (B 7) apply on the ambient-air-to-water interface *S*_0_ and on the totality of immersed stationary rigid-body surfaces *S*_*b*_, including the sea bed (figure 2):

B 7 $${\left.\left(-\omega^{2}+g\frac{\partial }{\partial z}\right)\left[\begin{array}{c} \phi_{s}\\ \varphi_{u,i}\\ \varphi_{p,k} \end{array}\right]\right|}_{S_{0}}=\left[\begin{array}{c} 0\\ 0\\ 0 \end{array}\right]\;\text{and}\;{\left.\frac{\partial }{\partial n}\left[\begin{array}{c} \phi_{s}\\ \varphi_{u,i}\\ \varphi_{p,k} \end{array}\right]\right|}_{S_{b}}=\left[\begin{array}{c} 0\\ 0\\ 0 \end{array}\right],$$

respectively.

Next, for completeness, we shall state the boundary conditions on the wave-radiating wetted surfaces *S*_*i*_=*S*_*u*,*i*_ of the immersed oscillating bodies and on the wave-radiating internal air–water interfaces *S*_*k*_=*S*_*p*,*k*_ of the OWCs (figure 2). It is convenient to define the two unions

B 8 $$S_{p}=\overset{N_{p}}{\underset{k=1}{⋃}}S_{p,k}\;\text{and}\;S_{u}=\overset{N_{u}}{\underset{i=1}{⋃}}S_{u,i}$$

as the totality of the two types of wave-radiating surfaces. On all OWC wave-radiating internal air–water interfaces *S*_*p*,*k*_, and on all oscillating rigid-body wetted surfaces *S*_*u*,*i*_, the following boundary conditions apply:

B 9 $${\left.\left(-\omega^{2}+g\frac{\partial }{\partial z}\right)\left[\begin{array}{c} \phi_{s}\\ \varphi_{u,i}\\ \varphi_{p,k} \end{array}\right]\right|}_{S_{p,k}}=\frac{-i\omega }{\rho }\left[\begin{array}{c} 0\\ 0\\ \delta_{k^{\prime }k} \end{array}\right]\;\text{and}\;{\left.\frac{\partial }{\partial n}\left[\begin{array}{c} \phi_{s}\\ \varphi_{u,i}\\ \varphi_{p,k} \end{array}\right]\right|}_{S_{u,i}}=\left[\begin{array}{c} 0\\ n_{i^{\prime }}\delta_{i^{\prime }i}\\ 0 \end{array}\right],$$

respectively, where *δ*_*k*′*k*_, as well as *δ*_*i*′*i*_, is the Kronecker delta, i.e. *δ*_*k*′*k*_=1 if *k*′=*k* and *δ*_*k*′*k*_=0 if *k*′≠*k*. Moreover, *n*_*i*_ is the *i*th component of the generalized unit-normal vector **n**=[*n*_1_ *n*_2_ *n*_3_ ⋯ *n*_*N*_u__]^T^ on the totality of all oscillating bodies' wetted surface *S*_*u*_.

**B.2. Kinetic–potential energy difference**

We are now ready to return to the problem of considering the difference between the kinetic energy and potential energy in the near-field region. The potential energy associated with the wave results from lifting water against gravity, and inside the *k*th OWC also against the dynamic air pressure *p*_*k*_. Referring to (B 3), (B 4), (B 8), (B 9) and figure 2, the time-average of the potential energy increases by an amount

B 10 $$\begin{array}{rl} W_{p} & =\iint_{S_{0}+S_{p}}E_{p}\;dS=\iint_{S_{0}}E_{p}\;dS+\sum_{k=1}^{N_{p}}\iint_{S_{k}}E_{p}\;dS\\ & =\iint_{S_{0}}\frac{\rho g|\eta {|}^{2}}{4}\;dS+\sum_{k=1}^{N_{p}}\iint_{S_{k}}\frac{\rho g|\eta_{k}{|}^{2}+p_{k}\eta_{k}^{*}+p_{k}^{*}\eta_{k}}{4}\;dS\\ & =\frac{\rho }{4}\iint_{S_{0}}\phi^{*}\frac{\partial \phi }{\partial z}\;dS+\frac{\rho }{4}\iint_{S_{p}}\left(\phi^{*}\frac{\partial \phi }{\partial z}+\frac{\partial \phi^{*}}{\partial z}\phi -\frac{g}{\omega^{2}}\frac{\partial \phi^{*}}{\partial z}\frac{\partial \phi }{\partial z}\right)dS \end{array}$$

due to the presence of the wave. Moreover, the time-average of the kinetic energy associated with the wave may be written as

B 11 $$\begin{array}{lll} W_{\text{k}} & = & \frac{\rho }{4}{∭}_{V}v^{*}\cdot v\text{d}V=\frac{\rho }{4}{∭}_{V}\nabla \phi^{*}\cdot \nabla \phi \text{d}V=\frac{\rho }{4}{∭}_{V}\nabla \cdot (\phi^{*}\nabla \phi )\text{d}V\\ & = & \frac{\rho }{4}∯(-n)\cdot \phi^{*}\nabla \phi \text{d}S=-\frac{\rho }{4}∯\phi^{*}\frac{\partial \phi }{\partial n}\text{d}S, \end{array}$$

noting that, as ∇^2^*ϕ*=0, then ∇*ϕ**⋅∇*ϕ*=∇⋅(*ϕ**∇*ϕ*)−*ϕ**∇^2^*ϕ*=∇⋅(*ϕ**∇*ϕ*), which enabled us to apply Gauss' divergence theorem. In (B 11), the volume integral is taken over the water volume as indicated in figure 2, while the surface integral is taken over the surface that encloses this water volume. This closed surface includes the envisaged, cylindrical, control surface $S_{\infty }$ of radius *r*. We may note that, because of the boundary condition ∂*ϕ*/∂*n*=0 on *S*_*b*_—cf. (B 7)—there is no contribution from surface *S*_*b*_ to the surface integral in (B 11). Moreover, noting that ∂/∂*n*=−∂/∂*z* on surface *S*_0_, we see that this surface contributes with equally large amounts to *W*_*k*_ and *W*_*p*_, and hence with zero contribution to *W*_*k*_−*W*_*p*_.

Thus, we may write

B 12 $$\begin{array}{rl} W_{k}-W_{p} & =\frac{\rho }{4}\iint_{S_{\infty }}\frac{\partial \phi^{*}}{\partial r}\phi \;dS-\frac{\rho }{4}\iint_{S_{u}}\frac{\partial \phi^{*}}{\partial n}\phi \;dS-\frac{\rho }{4}\iint_{S_{p}}\left(\phi^{*}-\frac{g}{\omega^{2}}\frac{\partial \phi^{*}}{\partial z}\right)\frac{\partial \phi }{\partial z}\;dS\\ & =\frac{\rho }{4}(I_{\infty }-I_{u}-I_{p}), \end{array}$$

where $I_{\infty }$, $I_{u}$ and $I_{p}$ represent the three integrals over the surfaces $S_{\infty }$, *S*_*u*_ and *S*_*p*_, respectively. Because *W*_k_−*W*_p_ is real, we may also write

B 13 $$\begin{array}{rl} W_{k}-W_{p} & =\frac{\rho }{4}(I_{\infty }-I_{u}-I_{p})=\frac{\rho }{4}{\left(I_{\infty }-I_{u}-I_{p}\right)}^{*}\\ & =\frac{\rho }{8}(I_{\infty }-I_{u}-I_{p}+I_{\infty }^{*}-I_{u}^{*}-I_{p}^{*}). \end{array}$$

Let us adopt the notation

B 14 $$T-V\equiv \lim_{kr\to \infty }(W_{k}-W_{p}),$$

and discuss separately, first, the radiation problem and, then, the scattering problem, for which all oscillators (cf. figure 2) are quiescent, i.e. **u**=**0** and **p**=**0**, and thus, *ϕ*_*r*_≡0, i.e. *ϕ*=*ϕ*_0_+*ϕ*_d_≡*ϕ*_*s*_. Finally, we consider the combined problem with incident wave *and* wave-radiating oscillations.

**B.3. Radiation problem**

For the radiation problem, for which there is no incident wave (*ϕ*_0_≡0) and hence also no diffracted wave (*ϕ*_d_=0), and so *ϕ*≡*ϕ*_*r*_, it is rather easy to show that the integrals over $S_{\infty }$ do not contribute to *T*−*V* . When kr→∞, then, according to (B 6), we have ∂*ϕ*_r_/∂*r*→−i*kϕ*_r_ and, consequently, $\partial \phi_{r}^{*}/\partial r\to ik\phi_{r}^{*}$. Thus, in this limit, there is no net contribution to *T*−*V* from the integrals over $S_{\infty }$, i.e. $I_{\infty }+I_{\infty }^{*}\to 0$ as kr→∞. This agrees with the fact that, in the far-field region, the surface density *E*_k_ of kinetic energy equals the surface density *E*_p_ of potential energy. They may, however, differ in the near-field region. Thus, we have from (B 13) and (B 14) that

B 15 $$T-V=-\lim_{kr\to \infty }\frac{\rho }{8}(I_{u}+I_{p}+I_{u}^{*}+I_{p}^{*}),$$

where now

B 16 $$I_{u}=\iint_{S_{u}}\frac{\partial \phi_{r}^{*}}{\partial n}\phi_{r}\;dS\;\text{and}\;I_{p}=\iint_{S_{p}}\left(\phi_{r}^{*}-\frac{g}{\omega^{2}}\frac{\partial \phi_{r}^{*}}{\partial z}\right)\frac{\partial \phi_{r}}{\partial z}\;dS.$$

Thus, by using (B 12)–(B 16), we may write

B 17 $$T-V=-\frac{\rho }{8}\iint_{S_{u}}\frac{\partial |\phi_{r}{|}^{2}}{\partial n}\;dS-\frac{\rho }{8}\iint_{S_{p}}\left(\frac{\partial |\phi_{r}{|}^{2}}{\partial z}-\frac{2g}{\omega^{2}}{\left|\frac{\partial \phi_{r}}{\partial z}\right|}^{2}\right)dS,$$

observing that $(\partial \phi_{r}^{*}/\partial n)\phi_{r}+(\partial \phi_{r}/\partial n)\phi_{r}^{*}=\partial (\phi_{r}^{*}\phi_{r})/\partial n$ and that $\phi_{r}^{*}\phi_{r}=\phi_{r}\phi_{r}^{*}=|\phi_{r}{|}^{2}$. (From (B 17), we may note that the sign in front of the integral over *S*_*p*_ disagrees with the corresponding incorrect sign in Falnes & McIver [10, eqn 87], because a complex-conjugation star in their eqn (81) is, incorrectly, placed on factor *v*_*z*_, rather than on factor *p*_*k*_. As a consequence, their eqn (96), which is incorrect, disagrees with our present new equation (B 25). The starting point for their derivation, their complex-valued eqn (81), was less obvious than our present, rather different, starting point, i.e. the real-valued equations (B 10) and (B 11).)

The velocity potential for the radiated wave is a superposition of waves radiated from the oscillating bodies and the OWCs (cf. (B 5))

B 18 $$\phi_{r}=\varphi_{u}^{T}\text{u}+\varphi_{p}^{T}\text{p}={\text{u}}^{T}\varphi_{u}+{\text{p}}^{T}\varphi_{p}.$$

Inserting this into (B 16) and then taking the sum, we get

B 19 $$I_{u}+I_{p}={\text{u}}^{†}{\text{M}}_{uu}\text{u}+{\text{p}}^{†}{\text{M}}_{pp}\text{p}+{\text{u}}^{†}{\text{M}}_{up}\text{p}+{\text{p}}^{†}{\text{M}}_{pu}\text{u},$$

where the four **M** matrices are given by

B 20 $$\begin{array}{rl} {\text{M}}_{uu} & =\iint_{S_{u}}\frac{\partial \varphi_{u}^{*}}{\partial n}\varphi_{u}^{T}\;dS+\iint_{S_{p}}\left(\varphi_{u}^{*}-\frac{g}{\omega^{2}}\frac{\partial \varphi_{u}^{*}}{\partial z}\right)\frac{\partial \varphi_{u}^{T}}{\partial z}\;dS\\ & =\iint_{S_{u}}\frac{\partial \varphi_{u}^{*}}{\partial n}\varphi_{u}^{T}\;dS=\frac{\text{Z}}{-i\omega \rho }, \end{array}$$

B 21 $$\begin{array}{rl} {\text{M}}_{pp} & =\iint_{S_{u}}\frac{\partial \varphi_{p}^{*}}{\partial n}\varphi_{p}^{T}\;dS+\iint_{S_{p}}\left(\varphi_{p}^{*}-\frac{g}{\omega^{2}}\frac{\partial \varphi_{p}^{*}}{\partial z}\right)\frac{\partial \varphi_{p}^{T}}{\partial z}\;dS\\ & =\iint_{S_{p}}\left(\varphi_{p}^{*}-\frac{g}{\omega^{2}}\frac{\partial \varphi_{p}^{*}}{\partial z}\right)\frac{\partial \varphi_{p}^{T}}{\partial z}\;dS=\frac{\text{Y}}{-i\omega \rho }, \end{array}$$

B 22 $$\begin{array}{rl} {\text{M}}_{up} & =\iint_{S_{u}}\frac{\partial \varphi_{u}^{*}}{\partial n}\varphi_{p}^{T}\;dS+\iint_{S_{p}}\left(\varphi_{u}^{*}-\frac{g}{\omega^{2}}\frac{\partial \varphi_{u}^{*}}{\partial z}\right)\frac{\partial \varphi_{p}^{T}}{\partial z}\;dS\\ & =\iint_{S_{u}}\frac{\partial \varphi_{u}^{*}}{\partial n}\varphi_{p}^{T}\;dS=\frac{{\text{H}}_{up}}{-i\omega \rho } \end{array}$$

B 23 $$\begin{array}{rl} \;\text{and}\;{\text{M}}_{pu} & =\iint_{S_{u}}\frac{\partial \varphi_{p}^{*}}{\partial n}\varphi_{u}^{T}\;dS+\iint_{S_{p}}\left(\varphi_{p}^{*}-\frac{g}{\omega^{2}}\frac{\partial \varphi_{p}^{*}}{\partial z}\right)\frac{\partial \varphi_{u}^{T}}{\partial z}\;dS\\ & =\iint_{S_{p}}\left(\varphi_{p}^{*}-\frac{g}{\omega^{2}}\frac{\partial \varphi_{p}^{*}}{\partial z}\right)\frac{\partial \varphi_{u}^{T}}{\partial z}\;dS=\frac{{\text{H}}_{pu}}{-i\omega \rho }. \end{array}$$

Here we have made use of the homogeneous boundary conditions for the radiated wave listed in the pair of equations (B 9)—cf. also (B 5). Moreover, following Falnes [35, §7.2], we have introduced the four complex radiation-parameter matrices **Z**, **Y**, **H**_*up*_ and **H**_*pu*_, which satisfy the reciprocity relations

B 24 $${\text{Z}}^{T}=\text{Z}=\text{R}+i\text{X},\;{\text{Y}}^{T}=\text{Y}=\text{G}+i\text{B}\;\text{and}\;-{\text{H}}_{pu}^{T}={\text{H}}_{up}\equiv \text{H}=\text{C}+i\text{J},$$

where the real matrices **R** and **G** represent radiation damping for the oscillating bodies and the OWCs, respectively [10, eqns 55–56]. As will appear explicitly from (B 25), the radiation-reactance matrix **X**=*ω***m**, as well as the added-mass matrix **m**, represents reactive energy corresponding to unbalance between kinetic energy and potential energy in the near-field part of the water surrounding the oscillating bodies. The real matrix **B** plays a corresponding role for the OWCs. The complex matrices **H**_*up*_ and **H**_*pu*_, as well as the real matrices **J** and **C**, represent hydrodynamic coupling between the oscillating bodies and the OWCs.

By using (B 20)–(B 23) in (B 19), insertion into (B 15) gives

B 25 $$\begin{array}{rl} T-V & =-\left(\frac{\rho }{8}\right)({\text{u}}^{†}{\text{M}}_{uu}\text{u}+{\text{p}}^{†}{\text{M}}_{pp}\text{p}+{\text{u}}^{†}{\text{M}}_{up}\text{p}+{\text{p}}^{†}{\text{M}}_{pu}\text{u})\\ & \;-\left(\frac{\rho }{8}\right){\left({\text{u}}^{†}{\text{M}}_{uu}\text{u}+{\text{p}}^{†}{\text{M}}_{pp}\text{p}+{\text{u}}^{†}{\text{M}}_{up}\text{p}+{\text{p}}^{†}{\text{M}}_{pu}\text{u}\right)}^{*}\\ & =-\left(\frac{\rho }{8}\right)({\text{u}}^{†}{\text{M}}_{uu}\text{u}+{\text{p}}^{†}{\text{M}}_{pp}\text{p}+{\text{u}}^{†}{\text{M}}_{up}\text{p}+{\text{p}}^{†}{\text{M}}_{pu}\text{u})\\ & \;-\left(\frac{\rho }{8}\right)({\text{u}}^{†}{\text{M}}_{uu}^{†}\text{u}+{\text{p}}^{†}{\text{M}}_{pp}^{†}\text{p}+{\text{p}}^{†}{\text{M}}_{up}^{†}\text{u}+{\text{u}}^{†}{\text{M}}_{pu}^{†}\text{p})\\ & =[{\text{u}}^{†}(\text{Z}-{\text{Z}}^{†})\text{u}+{\text{p}}^{†}(\text{Y}-{\text{Y}}^{†})\text{p}+{\text{u}}^{†}({\text{H}}_{up}-{\text{H}}_{pu}^{†})\text{p}+{\text{p}}^{†}({\text{H}}_{pu}-{\text{H}}_{up}^{†})\text{u})]/(8i\omega )\\ & =\left({\text{u}}^{†}\text{X}\text{u}+{\text{p}}^{†}\text{B}\text{p}\right)/\left(4\omega \right)+\left({\text{u}}^{†}\text{C}\text{p}-{\text{p}}^{†}{\text{C}}^{T}\text{u}\right)/\left(4i\omega \right)\\ & =\frac{1}{4\omega }[{\text{u}}^{†}\;-{\text{p}}^{†}]\left[\begin{array}{cc} \text{X} & i\text{C}\\ -i{\text{C}}^{T} & \text{B} \end{array}\right]\left[\begin{array}{c} \text{u}\\ -\text{p} \end{array}\right]\\ & =\left({\text{u}}^{†}\text{X}\text{u}+{\text{p}}^{†}\text{B}\text{p}+2\;\text{Im}\{{\text{u}}^{†}\text{C}\text{p}\}\right)/\left(4\omega \right). \end{array}$$

(Note that we here, in the fourth line, have transposed all four matrix products, which are scalars.) This result (B 25) is a generalization of (B 1), which clearly reveals that the added-mass matrix **m**=**X**/*ω* is directly related to, not the kinetic energy alone, but the difference between the kinetic energy and the potential energy in the near-field region of the radiating system. This generalized expression (B 25) contains the three real matrices **X**, **B** and **C**. By making use of reciprocity relations (B 24) and equations (B 20)–(B 23), we may express these three matrices, explicitly, in terms of velocity-potential coefficients for the radiated wave, as follows:

B 26 X=ωm=Im{Z}=(Z−Z∗)/(2i)=(Z−Z†)/(2i)=−ωρ2∬Su∂φu∗∂nφuT dS−ωρ2∬Suφu∗∂φuT∂n dS=−ωρ2∬Su∂(φu∗φuT)∂n dS=−ωρ2∬Su∂(φuφu†)∂n dS,

B 27 B=Im{Y}=(Y−Y∗)/(2i)=(Y−Y†)/(2i)=−ωρ2∬Spφp∗−gω2∂φp∗∂z∂φpT∂z dS−ωρ2∬Sp∂φp∗∂zφpT−gω2∂φpT∂zdS=−ωρ2∬Sp∂(φp∗φpT)∂z−2gω2∂φp∗∂z∂φpT∂zdS=−ωρ2∬Sp∂(φpφp†)∂z−2gω2∂φp∂z∂φp†∂zdS

B 28 andC=Re{H}≡Re{Hup}=(Hup+Hup∗)/2=(Hup−Hpu†)/2=−iωρ2∬Su∂φu∗∂nφpT dS−iωρ2∬Sp∂φu∗∂zφpT−gω2∂φpT∂zdS=iωρ2∬Su∂φu∂nφp† dS+iωρ2∬Sp∂φu∂zφp†−gω2∂φp†∂zdS.

We may note from (B 20)–(B 23) and (B 26)–(B 28) that the complex radiation-parameter matrices **Z**, **Y**, **H**_*up*_ and **H**_*pu*_, as well as the real matrices **X**, **B** and **C**, may be represented by integrals over the wave-radiating surfaces *S*_*u*_ and/or *S*_*p*_. Observe that (B 26) is a generalization of Falnes [9, eqn 28] to the case when the oscillation system is composed of, in addition to immersed bodies, also OWCs. Our present three equations (B 26)–(B 28) provide explicit expressions for matrices **X**, **B** and **C**, which are three of six real matrices that appear on the r.h.s. of the three equations (B 24). The three remaining ones are **R**, **G** and **J**, which may be explicitly expressed by integrals over the envisaged far-field control surface $S_{\infty }$, where the integrand contains terms of velocity-potential coefficients for the radiated wave (e.g. [10, eqns 61–64]). To the best of the authors' knowledge, the formulae (B 27) and (B 28) are completely new results that have not been published before.

**B.4. Scattering problem**

For the case of a quiescent oscillating system, that is [**u**^T^ **p**^T^]=**0**, there is no radiated wave, *ϕ*_r_=0. Hence, we have no other wave than the scattered one, which is a superposition of the incident and the diffracted waves. This means that (B 5), for this case, simplifies to *ϕ*=*ϕ*_*s*_=*ϕ*_0_+*ϕ*_d_. If we make this substitution into $I_{u}$ and $I_{p}$ in (B 12), and use the first one of the three boundary conditions listed in both of equations (B 9), we find that $I_{u}=0$ and $I_{p}=0$ for the scattering problem. For this case the ‘near-field’ boundary conditions on *S*_*p*_ and *S*_*u*_ are homogeneous. Thus from (B 13) and (B 14), we have

B 29 $$T-V\equiv \lim_{kr\to \infty }\frac{\rho }{8}(I_{\infty }+I_{\infty }^{*})$$

for the scattering problem. We may remark that this equation is quite different from the corresponding equation (B 15) for the radiation problem.

For the radiation problem, we found it rather easy to show that $I_{\infty }+I_{\infty }^{*}\to 0$ as kr→∞. We shall see that the same holds for the scattering problem, provided that we let the radius *r* of the envisaged control cylinder run to infinity through a set of discrete values that are separated by a multiple of a quarter wavelength. The proof, which is sketched below, is, however, somewhat more intricate for the scattering problem than for the radiation problem. We shall conclude that the scattered wave does not contribute to *T*−*V* , except for a small standing-wave contribution, which averages to zero over every half-wavelength increment of the radius *r* of the cylindrical control surface $S_{\infty }$.

To evaluate the quantity $I_{\infty }+I_{\infty }^{*}$ for the scattering problem, we find from (B 12) that

B 30 $$I_{\infty }=\iint_{S_{\infty }}\frac{\partial \phi^{*}}{\partial r}\phi \;dS=\iint_{S_{\infty }}\frac{\partial \phi_{s}^{*}}{\partial r}\phi_{s}\;dS=\iint_{S_{\infty }}\frac{\partial {{(\phi }_{0}+\phi_{d})}^{*}}{\partial r}(\phi_{0}+\phi_{d})\;dS.$$

For convenience, we write this as

B 31 $$I_{\infty }=I_{\infty ,00}+I_{\infty ,0d}+I_{\infty ,dd},$$

where

B 32 $$\begin{array}{c} I_{\infty ,0d}=\iint_{S_{\infty }}\left(\frac{\partial \phi_{0}^{*}}{\partial r}\phi_{d}+\frac{\partial \phi_{d}^{*}}{\partial r}\phi_{0}\right)dS, \end{array}$$

B 33 $$\begin{array}{c} I_{\infty ,00}=\iint_{S_{\infty }}\frac{\partial \phi_{0}^{*}}{\partial r}\phi_{0}\;dS\;\text{and}\;I_{\infty ,dd}=\iint_{S_{\infty }}\frac{\partial \phi_{d}^{*}}{\partial r}\phi_{d}\;dS. \end{array}$$

We shall here assume that the mean sea surface is the plane *z*=0, and that the sea bed, at least outside the envisaged control cylinder $S_{\infty }$, is at the plane *z*=−*h*. For an incident plane wave propagating at an angle *β* with respect to the *x*-axis, the velocity potential is, for all coordinates (x,y,z)=(rcos⁡θ,rsin⁡θ,z) in the water, given by

B 34 $$\phi_{0}=a_{0}e(kz)\;e^{-ikr\cos(\theta -\beta )},$$

where e(kz)=cosh⁡(kz+kh)/cosh⁡(kh), and where *a*_0_=(−*g*/i*ω*)*A*. Here *A* is the complex amplitude of the incident wave elevation at the origin, *r*=0. Moreover, in the far-field region, the diffracted wave is given, asymptotically, by

B 35 $$\phi_{d}\to s_{d}(\theta )e(kz){\left(kr\right)}^{-1/2}\;e^{-ikr}\;\text{as}\;kr\to \infty ,$$

where *s*_d_(*θ*) is a complex function that determines, in the far field, not only the amplitude and the phase of the wave, but also their directional variation. When kr→∞, then, according to (B 6) or (B 35), we have ∂*ϕ*_d_/∂*r*→−i*kϕ*_d_ and, consequently, $\partial \phi_{d}^{*}/\partial r\to ik\phi_{d}^{*}$. Thus, in this limit, $I_{\infty ,dd}+I_{\infty ,dd}^{*}\to 0$ as kr→∞. Furthermore, from (B 34) we find that, everywhere in the water, and consequently also on $S_{\infty }$, we have $\partial \phi_{0}/\partial r=-ik\cos(\theta -\beta )\phi_{0}$ and, thus, $\partial \phi_{0}^{*}/\partial r=ik\cos(\theta -\beta )\phi_{0}^{*}$. If we use this information in (B 33), we may conclude that $I_{\infty ,00}+I_{\infty ,00}^{*}=0$, and hence,

B 36 $$I_{\infty }+I_{\infty }^{*}=I_{\infty ,0d}+I_{\infty ,0d}^{*}=I_{\infty ,0d}^{\prime }+I_{\infty ,0d}^{\prime *}$$

for the scattering problem. Here $I_{\infty ,0d}^{\prime }$ is derived and defined below (see (B 38)).

What now remains is a more difficult task, namely, to discuss the limit of $I_{\infty ,0d}+I_{\infty ,0d}^{*}$ as kr→∞. Using the product rule, we find from (B 32) that

B 37 $$I_{\infty ,0d}+I_{\infty ,0d}^{*}=\iint_{S_{\infty }}\frac{\partial }{\partial r}(\phi_{0}^{*}\phi_{d}+\phi_{0}\phi_{d}^{*})\;dS=\iint_{S_{\infty }}\frac{\partial }{\partial r}(\phi_{0}^{*}\phi_{d})\;dS+c.c.,$$

where c.c. denotes the complex conjugate of the preceding term, namely,

B 38 $$\iint_{S_{\infty }}\frac{\partial }{\partial r}(\phi_{0}^{*}\phi_{d})\;dS\equiv I_{\infty ,0d}^{\prime },$$

say. (Note that although $I_{\infty ,0d}^{\prime }$ and $I_{\infty ,0d}$ may be unequal, they have equal real parts.) If we assume that the envisaged control surface $S_{\infty }$ is cylindrical with radius r→∞, d*S*=*r* d*θ* d*z*, where *θ* and *z* run from *β* to *β*+2*π* and from −*h* to 0, respectively. From expressions (B 34) and (B 35), we find that in (B 38) the integrand $\to -ik(1-\cos\varphi )\phi_{0}^{*}\phi_{d}$, where *φ*=*θ*−*β* has been chosen as a new integration variable instead of *θ*. Concerning the integral over *z*, we here just need to state that it is finite, real, positive and equal to *C*_*z*_, say. Then we have, as kr→∞,

B 39 $$I_{\infty ,0d}^{\prime }\to -ia_{0}^{*}C_{z}\int_{0}^{2\pi }{\left(kr\right)}^{1/2}(1-\cos\varphi )s_{d}(\varphi +\beta )\;e^{-ikr(1-\cos\varphi )}\;d\varphi .$$

Because kr→∞, this integral may be evaluated by the ‘method of stationary phase’. We find that (e.g. [35, p. 100, eqn 4.288])

B 40 $$\begin{array}{rl} I_{\infty ,0d}^{\prime } & \to -ia_{0}^{*}C_{z}2{\left(\pi \right)}^{1/2}s_{d}(\pi +\beta )(1+i)\;e^{-i2kr}\\ & =a_{0}^{*}C_{z}2{\left(\pi \right)}^{1/2}s_{d}(\pi +\beta )(1-i)\exp\{-i2kr\}\\ & =|a_{0}|C_{z}{\left(8\pi \right)}^{1/2}|s_{d}(\pi +\beta )|\exp\left\{-i\left(2kr+\frac{\pi }{4}+\alpha_{0}-\sigma_{d}\right)\right\}, \end{array}$$

where $\alpha_{0}=\arg\{a_{0}\}$ and $\sigma_{d}=\arg\{s_{d}(\pi +\beta )\}$.

To summarize, from (B 29)–(B 32) and (B 37)–(B 40), we have

B 41 $$T-V=\lim_{kr\to \infty }\frac{\rho }{8}(I_{\infty }+I_{\infty }^{*})=\lim_{kr\to \infty }\frac{\rho }{8}(I_{\infty ,0d}^{\prime }+I_{\infty ,0d}^{\prime *}),$$

where

B 42 $$I_{\infty ,0d}^{\prime }+I_{\infty ,0d}^{\prime *}=\left(\frac{8}{\rho }\right)\Delta_{kp}\cos\left(2kr+\frac{\pi }{4}+\alpha_{0}-\sigma_{d}\right)$$

and

B 43 $$\Delta_{kp}=\rho |a_{0}|C_{z}{\left(\frac{\pi }{2}\right)}^{1/2}|s_{d}(\pi +\beta )|.$$

Hence, we have arrived at

B 44 $$T-V=\lim_{kr\to \infty }\Delta_{kp}\cos\left(2kr+\frac{\pi }{4}+\alpha_{0}-\sigma_{d}\right).$$

This means that, for sufficiently large, increasing values of *kr*, the energy difference *T*−*V* varies between Δ_*kp*_ and −Δ_*kp*_. We note that *T*−*V* =0 for the discrete values

B 45 $$kr=kr_{\nu }\equiv \frac{\nu \pi }{2}+\frac{\pi }{8}-\frac{\alpha_{0}}{2}+\frac{\sigma_{d}}{2},$$

where *ν* is a sufficiently large integer. The distance *r*_*ν*+1_−*r*_*ν*_ is only one-quarter of a wavelength. This far-field spatial variation is just an interference effect because of waves—such as our present incident wave *ϕ*_0_ and diffracted wave *ϕ*_d_—propagating in different directions. For instance, if a plane wave is totally reflected, a standing wave with nodes and antinodes results. In the nodes, the potential energy density is zero, but it is rather large in the antinodes. Averaged over half a wavelength, the potential energy and the kinetic energy are equally large. As is obvious from (B 44), if we average *T*−*V* over a radial distance Δ*r*=*π*/*k*=*r*_*ν*+2_−*r*_*ν*_, the result is zero.

**B.5. Combined full problem**

So far, we have found that, in time average, and integrated over the total near-field region, the *pure* scattering problem contributes essentially nothing to the difference between kinetic energy and potential energy. Having discussed the pure radiation problem and the pure scattering problem separately, let us, finally, consider the combined problem, with *ϕ*=*ϕ*_0_+*ϕ*_d_+*ϕ*_r_=*ϕ*_s_+*ϕ*_r_. As the radiated wave *ϕ*_r_ and the diffracted wave *ϕ*_d_ satisfy the same radiation condition (B 6) at infinity, the discussion of $I_{\infty }+I_{\infty }^{*}$ for the scattering problem is directly applicable also for the combined problem.

We found above that the two integrals ℐ_*u*_ and ℐ_*p*_ defined in (B 12) vanish for the scattering problem, while they for the radiation problem have finite values as given by (B 16). For the combined full problem, there are additional contributions ℐ_*u*,*rs*_ and ℐ_*p*,*rs*_, say. The corresponding two integrands contain two-factor products of *ϕ*_*s*_ and *ϕ*_r_ (or their derivatives), products of the type ${\left(\phi_{s}+\phi_{r}\right)}^{*}(\phi_{s}+\phi_{r})=\phi_{s}^{*}\phi_{s}+\phi_{s}^{*}\phi_{r}+\phi_{r}^{*}\phi_{s}+\phi_{r}^{*}\phi_{r}$. Here, the first and fourth terms correspond to the scattering problem and the radiation problem, respectively, while the remaining terms $\left(\phi_{s}^{*}\phi_{r}+\phi_{r}^{*}\phi_{s}\right)$ will be our present consideration in the following discussion, unique for the combined full problem.

Then, in accordance with definitions (B 12) and boundary conditions (B 9), we have

B 46 $$\begin{array}{rl} I_{u.rs} & =\iint_{S_{u}}\left(\frac{\partial \phi_{s}^{*}}{\partial n}\phi_{r}+\frac{\partial \phi_{r}^{*}}{\partial n}\phi_{s}\right)dS=\iint_{S_{u}}\frac{\partial \phi_{r}^{*}}{\partial n}\phi_{s}\;dS\\ & =\frac{1}{-i\omega \rho }\sum_{i=1}^{N_{u}}\iint_{S_{u,i}}{\left(n_{i}u_{i}\right)}^{*}p_{s}\;dS=\frac{1}{i\omega \rho }\sum_{i=1}^{N_{u}}F_{e,i}u_{i}^{*}=\frac{1}{i\omega \rho }{\text{F}}_{e}^{T}{\text{u}}^{*} \end{array}$$

and

B 47 $$\begin{array}{rl} I_{p,rs} & =\iint_{S_{p}}\left\{\left(\phi_{s}^{*}-\frac{g}{\omega^{2}}\frac{\partial \phi_{s}^{*}}{\partial z}\right)\frac{\partial \phi_{r}}{\partial z}+\left(\phi_{r}^{*}-\frac{g}{\omega^{2}}\frac{\partial {\phi_{r}}^{*}}{\partial z}\right)\frac{\partial \phi_{s}}{\partial z}\right\}dS\\ & =\iint_{S_{p}}\left(\phi_{r}^{*}-\frac{g}{\omega^{2}}\frac{\partial \phi_{r}^{*}}{\partial z}\right)\frac{\partial \phi_{s}}{\partial z}\;dS=\frac{1}{i\omega \rho }\sum_{k=1}^{N_{p}}Q_{e,k}p_{k}^{*}=\frac{1}{i\omega \rho }{\text{Q}}_{e}^{T}{\text{p}}^{*}, \end{array}$$

where, in (B 46), *p*_*s*_=[−i*ωρϕ*_*s*_]_*S*_*u*,*i*__ is the hydrodynamic pressure, *F*_e,*i*_ is the excitation force, and *n*_*i*_*u*_*i*_=[∂*ϕ*_r_/∂*n*]_*S*_*u*,*i*__ is the normal component of the wetted-body-surface velocity for the *i*th oscillating-body mode; see the second one of the two boundary conditions (B 9). Further, in (B 47),

B 48 $$Q_{e,k}=\iint_{S_{p,k}}\frac{\partial \phi_{s}}{\partial z}\;dS\;\text{and}\;p_{k}=-i\omega \rho {\left[\phi_{r}-\frac{g}{\omega^{2}}\frac{\partial \phi_{r}}{\partial z}\right]}_{S_{p,k}}$$

are the excitation volume flow and the dynamic air pressure for the *k*th OWC, respectively; see the first one of the two boundary conditions (B 9).

With (B 46) and (B 47), it follows from (B 12)–(B 14) that, associated with absorption of power from the incident wave, there is an additional contribution of

B 49 (T−V)rs=−18iω(FeTu∗−Fe†u+QeTp∗−Qe†p)=−14ω Im{FeTu∗+QeTp∗}

to the difference between kinetic and potential energy stored in the water surrounding the WEC array. This contribution is adding to the energy difference which is quantified by (B 25), and which is applicable to a situation when the WEC array is performing forced oscillation in the absence of an incident wave.
